# Exploring PadR Proteins for Artificial Enzyme Design

**DOI:** 10.1002/cbic.70308

**Published:** 2026-04-28

**Authors:** Bart Brouwer, Andy-Mark W. H. Thunnissen, Henriette J. Rozeboom, Gerard Roelfes

**Affiliations:** ^1^ Stratingh Institute for Chemistry University of Groningen Groningen Netherlands; ^2^ Groningen Biomolecular Sciences and Biotechnology Institute University of Groningen Groningen Netherlands

**Keywords:** artificial enzyme, biocatalysis, noncanonical amino acid, PadR‐s2, protein scaffold

## Abstract

The development of artificial enzymes through incorporation of new‐to‐nature catalytic functionality into protein scaffolds has emerged as a powerful approach to expand the biocatalytic repertoire. Inspired by the success of *Lactococcal* multidrug resistance regulator (LmrR), a transcriptional regulator protein, whose unique scaffold has been used for the design of a range of artificial enzymes, we performed a bioinformatics study in an effort to expand the scope of protein scaffolds for artificial enzyme design with other LmrR‐like proteins. LmrR belongs to the phenolic acid decarboxylase transcriptional regulator (PadR) subfamily 2 (PadR‐s2) and exhibits an unusual open pore with promiscuous binding capabilities. Using genome mining and homology modeling, we identified six previously uncharacterized PadR‐s2 proteins and experimentally evaluated them as protein scaffolds for the design of artificial Friedel–Crafts (FC) alkylases. Two of the candidates, *Lactococcus fujiensis* (LCf) PadR and *Brachyspirahampsonii* (Bh) PadR, could be applied in the iminium‐promoted FC‐alkylation using genetically incorporated noncanonical amino acids p‐aminophenylalanine or 3‐aminotyrosine as catalytic residues. Interestingly, contrary to homology models, AlphaFold predictions of the PadR‐s2 candidates and X‐ray crystallography of BhPadR and a variant incorporating 3‐aminotyrosine revealed closed‐pore structures. Our findings thus demonstrate that an open‐pore structure like LmrR is not a prerequisite for designing artificial FC‐alkylases and introduce two new PadR‐s2 scaffolds for future application.

## Graphical abstract

Inspired by the *Lactococcal* multidrug resistance regulator (LmrR) protein scaffold, new phenolic acid decarboxylase transcriptional regulator (PadR) proteins were identified and evaluated as scaffolds for artificial enzyme design. Variants incorporating noncanonical amino acids bearing a primary amine were able to partake in iminium catalysis. Structural analysis revealed a closed‐pore structure, demonstrating that an open‐pore architecture, as found in LmrR, is not strictly required for creating PadR‐based artificial enzymes.

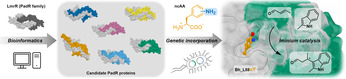

## 1 Introduction

**FIGURE 1:**
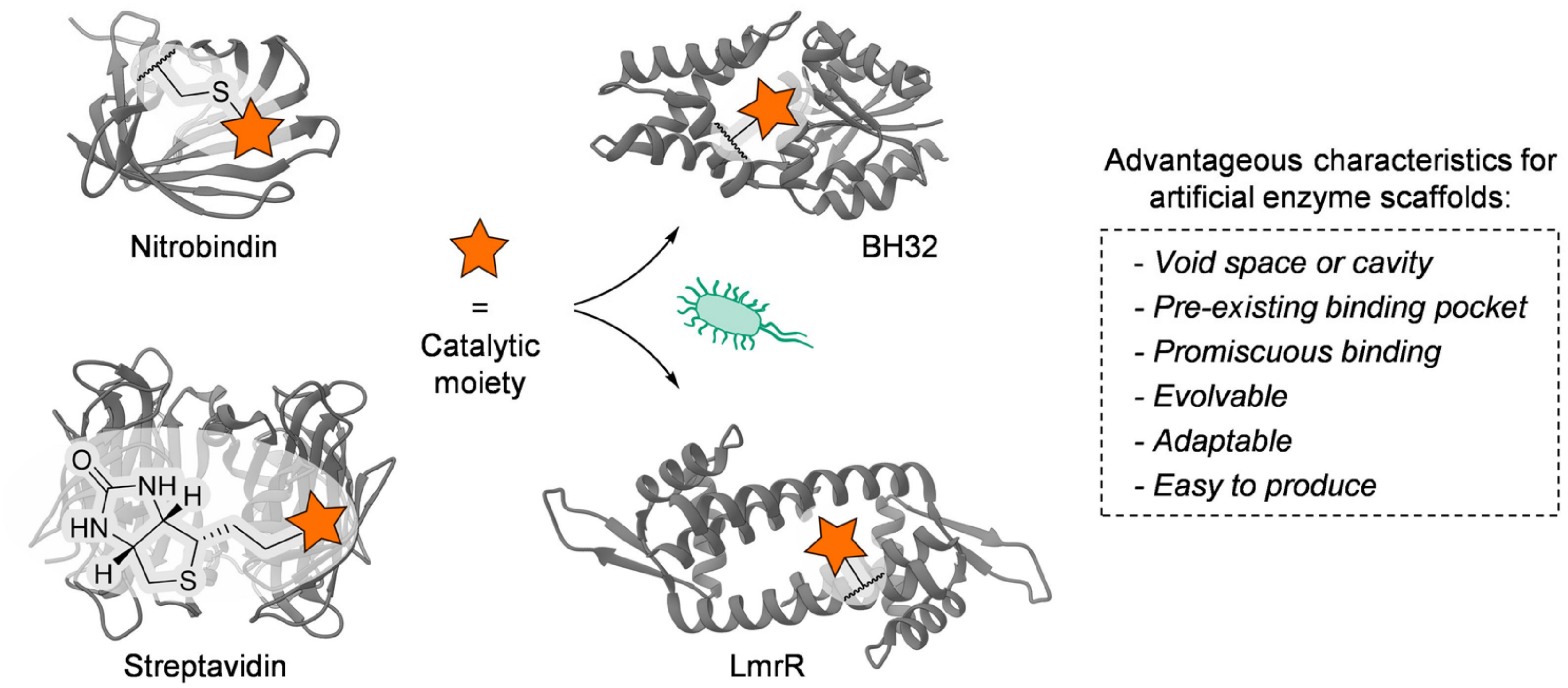
Examples of protein scaffolds used for the design of artificial enzymes. Nitrobindin (PDB 3WJC) [14], depicting the introduction of a catalytic moiety into its cavity via cysteine conjugation; Streptavidin (PDB 6GH7) [15], exploiting its affinity for biotin to introduce a biotinylated catalytic moiety; BH32 (PDB 8BP1) [16] and LmrR (PDB 3F8B) [17], introducing a catalytic moiety via genetic incorporation of an ncAA.

The ability to engineer and evolve natural enzymes has revolutionized the application of biocatalysts for organic synthesis [1, 2, 3, 4]. However, the defined set of chemical functionalities available to natural enzymes can hamper their evolution toward new activation modes, leaving their catalytic repertoire to be more limited compared to conventional chemical catalysts. To overcome this challenge, recent years have seen a surge in campaigns to develop artificial enzymes, in which proteins are endowed with new‐to‐nature chemical groups, allowing catalytic activation strategies not generally available to nature [5, 6, 7, 8]. Approaches to introduce such abiological chemistries into proteins include covalent anchoring [9, 10, 11], noncovalent binding or anchoring of unnatural cofactors [9, 12, 13], and genetic encoding of noncanonical amino acids (ncAAs) (Figure 1) [18, 19]. The latter is particularly powerful in this regard, as it allows for the incorporation of new chemical functionality at any desired position in virtually any protein scaffold. Using these methods, rudimentary enzymes can be created that exhibit a basal level of activity in new‐to‐nature transformations, which can subsequently be improved via directed evolution.

The choice of protein scaffold is of key importance in the design of artificial enzymes, as it determines the chemical microenvironment for catalysis, which plays a critical role in modulating the reactivity of the introduced catalytic moiety and can aid in activation or binding of desired substrates. Furthermore, the choice of protein scaffold ultimately also affects other functional properties such as stability, conformational flexibility, and ease of production. While there is no universal approach to what makes a good protein scaffold for artificial enzyme design, there are certain properties that are deemed to be advantageous. For example, the presence of a pre‐existing binding pocket or void space that can serve as a starting point for the creation of a new active site or as an anchoring site to introduce a new catalytic moiety, but also the adaptability and evolvability of the scaffold, that being the possibility to incorporate varying catalytic moieties and, subsequently, mutations to optimize the created rudimentary enzymes [20, 21]. Examples of successfully applied protein scaffolds (Figure 1) include Nitrobindin, harboring a void space that has been exploited to conjugate a variety of non‐natural metal catalysts [9, 21]; Streptavidin, whose native affinity for biotin has been used to introduce a wide range of abiological chemistries through biotinylated catalytic moieties [15, 22, 23]; and also computationally designed proteins such as BH32, found to be adaptable toward different transformations by exploiting a noncanonical organocatalytic residue [16, 24, 25]. Our group has mainly employed *Lactococcal* multidrug resistance regulator (LmrR), a small homodimeric protein harboring an open pore that exhibits promiscuous binding capabilities owing to its native function [17]. As demonstrated by the range of artificial enzymes created through incorporation of different catalytic moieties into LmrR, its open pore has served as an outstanding starting point for the creation of novel active sites [10, 18, 26, 27, 28].

The creation of artificial enzymes that exhibit basal activities toward desired transformations, however, remains difficult, as distinct properties are often required for catalysis. The availability of multiple protein scaffolds with varying characteristics can aid in this regard, as it increases the chances of finding the right microenvironment for catalysis. Approaches to identify new scaffolds include the identification of proteins with known binding affinities for target substrates, genome mining to search for homologs of established scaffolds, and exploration of protein families with similar functions as previously successful scaffolds [29, 30, 31]. An example of the latter is the multidrug resistance regulators (MDRs) of the tetracyclin repressor (TetR) family, which, similar to LmrR, harbor promiscuous binding pockets related to their native function [30]. Recent work, employing one of these TetR family proteins as a scaffold in a designer boron enzyme, clearly demonstrated the significance of having multiple scaffolds available for the design of new artificial enzymes, as among the different MDRs tested, it was the only scaffold that provided the appropriate environment for the desired enantiodiscrimination in catalysis [32].

In this work, we aimed to further expand the available library of protein scaffolds for artificial enzyme design. Given the success of LmrR and its open pore in the construction of artificial enzymes, we hypothesized that homologs of LmrR with similar open‐pore structures but varying pore microenvironments could serve as valuable additional protein scaffolds for the construction of new artificial enzymes. To this end, we performed a bioinformatics study to explore the protein family of LmrR, involving mining and homology modeling of proteins from the phenolic acid decarboxylase transcriptional regulator (PadR) family in an effort to find new scaffolds with LmrR‐like structural characteristics. We identified and produced six previously uncharacterized PadR proteins, from which two could be used as scaffolds to construct artificial Friedel–Crafts (FC) alkylases employing genetically incorporated ncAAs as catalytic residues. Structural analysis showed that the new scaffolds lack the characteristic open pore of LmrR, demonstrating that this feature is not strictly required for creating PadR‐based artificial enzymes.

## 2 Results and Discussion

### 2.1 Mining New PadR Proteins

**FIGURE 2:**
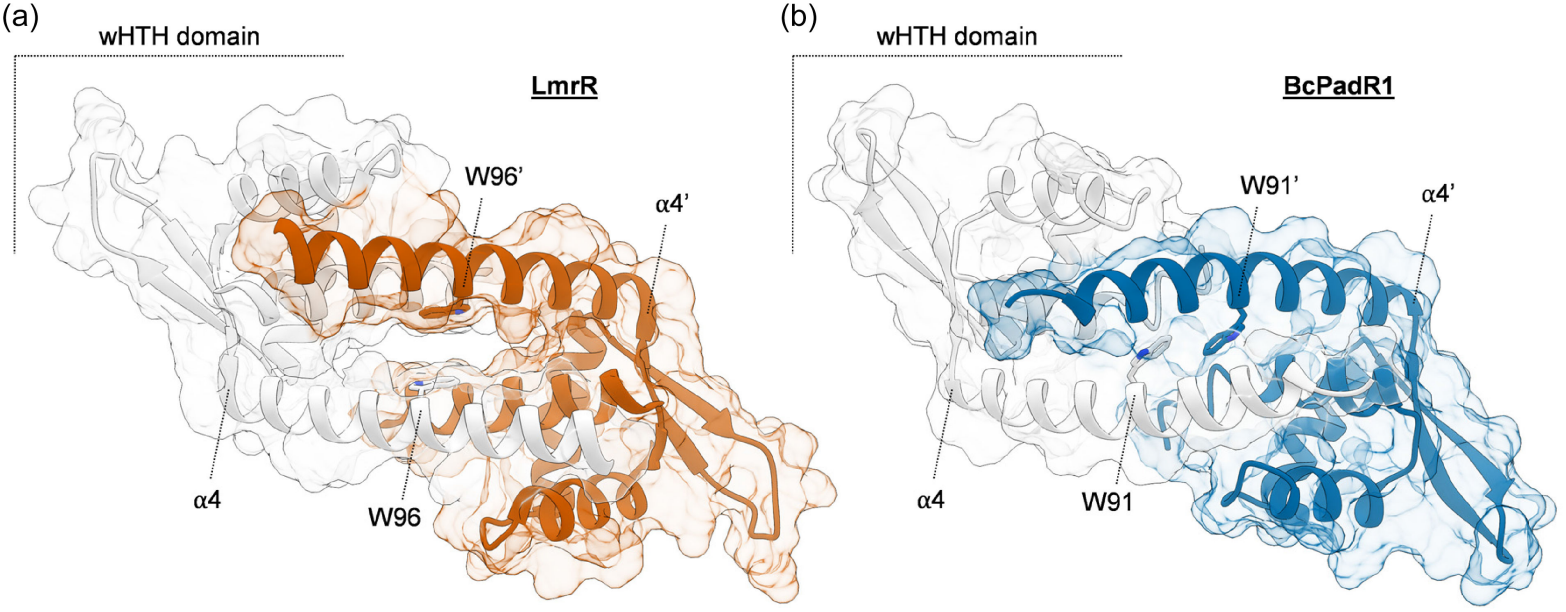
Crystal structures of two proteins from the PadR‐s2 family. (a) LmrR (PDB 3F8B), harboring an open pore at the dimeric interface [17]. (b) BcPadR1 (PDB 4ESB), exhibiting a closed dimeric interface [33]. One of the monomers of each structure is colored. The wHTH domain and the C‐terminal α4 helix containing a conserved tryptophan are annotated. The apostrophe indicates that the residue or helix resides in the dimer‐related subunit.

**FIGURE 3:**
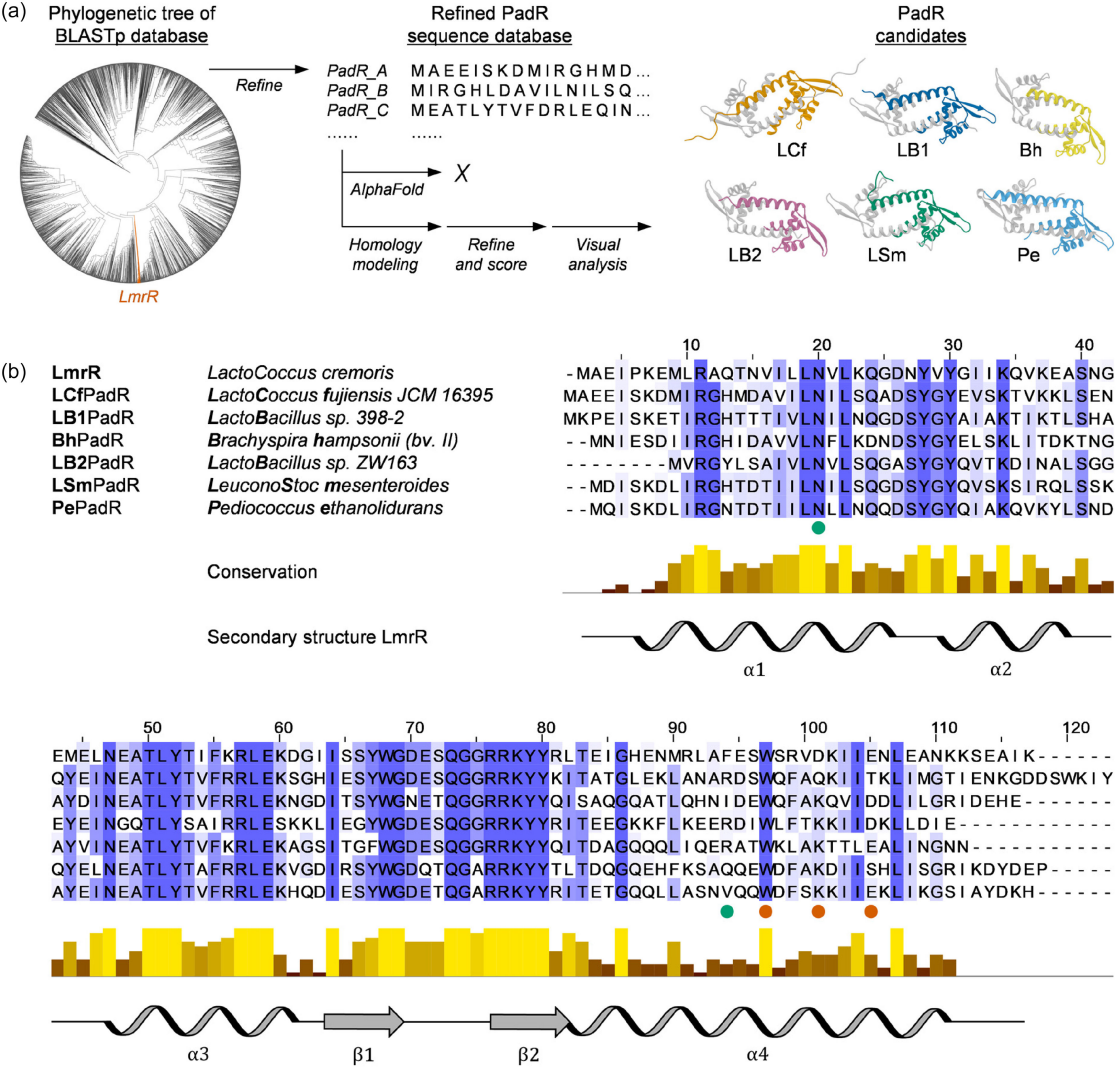
(a) Workflow used to identify six PadR candidates for experimental testing. The refined database of putative PadR sequences was generated by selecting sequences in close proximity to LmrR in phylogenetic trees constructed from a database of sequences obtained from a BLASTp using LmrR as query (see Section S1 for more details). AlphaFold [39, 40] predictions did not lead to open‐pore PadR structures. Homology models based on defined open‐pore LmrR and closed‐pore PadR‐s2 template crystal structures were constructed, refined through short 0.5 ns molecular dynamics simulations, and scored using Yasara (see Section S2 for more details) [41]. PadR candidates were selected after visual inspection of sequences for which homology models constructed based on the LmrR templates scored better than the homology models constructed based on the other PadR‐s2 templates. The displayed protein structures correspond to the highest‐scoring LmrR‐based homology models. (b) Multiple sequence alignment of LmrR and the six PadR candidates. Residues are colored in blue with intensity scaled according to percentage identity and conservation. Selected residues are marked with a green or orange dot to indicate their role in forming stabilizing interactions across the dimer interface in the crystal structure of Bh (Figure 4). Below the alignment, overall sequence conservation, and the experimentally observed secondary structure of LmrR are displayed. Conservation is colored from low (brown) to fully conserved (yellow). Jalview and the built‐in ClustalO web service (with defaults) were used for multiple sequence alignments [42].

LmrR belongs to the PadR‐s2 subfamily, containing proteins that span ≈ 110 amino acids, comprising a characteristic winged helix‐to‐helix (wHTH) DNA‐binding domain and a C‐terminal helix (α4) involved in dimerization [17, 33, 34, 35]. The latter positions two tryptophans at the dimer interface (W96 and W96’), which, in the case of LmrR, can be involved in π‐stacking interactions with planar heterocyclic compounds (Figure 2a) [17, 36, 37, 38]. The characteristic open pore at the dimeric interface of LmrR appears to be an exclusive feature, as all other entries of PadR‐s2 protein structures in the protein data bank (PDB) to date exhibit a closed dimeric interface (Figure 2b). Given the unique structural feature of LmrR and its outstanding application as protein scaffold in the construction of artificial enzymes, we set out to identify other, uncharacterized PadR‐s2 proteins as potential new open‐pore scaffolds with different microenvironments. The amino acid sequence of LmrR was used as a query to perform a protein BLAST search and create a database of LmrR‐like proteins. Sequences with <25% identity to LmrR were discarded, and results were narrowed down to protein sequences of 99–130 amino acids in size in order to only target proteins from the PadR‐s2 subfamily [33, 34, 35]. The thus obtained database (3806 sequences) was used to create multiple phylogenetic trees and further narrowed down by only retaining clades positioned in close proximity to LmrR, leading to a refined database of 112 putative PadR sequences (Figure 3a; see Section S1 for more details). AlphaFold [39, 40] was used to predict the structures of these putative PadR proteins starting from their amino acid sequences. We found that all proteins were predicted to exhibit a closed dimeric interface, similar to most PadR‐s2 crystal structures. However, AlphaFold predictions of these proteins may be biased toward closed‐pore structures due to the presence of various closed‐pore PadR‐s2 family members in the PDB, and LmrR being the only open‐pore example. Furthermore, potential conformational dynamics are not sampled by these static structure predictions [43, 44], and similarly to LmrR, these PadR proteins might exhibit a degree of conformational flexibility [45, 46, 47]. In an alternative approach, the likelihood of the PadR sequences to exhibit similar structural characteristics as LmrR was assessed through homology modeling with Yasara (Figure 3a; see Section S2 for more details) [41]. Crystal structures of both LmrR and other PadR‐s2 proteins were used as templates, resulting in multiple 3D models per sequence, exhibiting the characteristic open‐pore structure for LmrR‐based models and the more closed‐pore structure for all other PadR‐s2‐based models. The obtained homology models were then refined by performing short 0.5 ns molecular dynamics simulations and scored on their model quality [41]. Sequences for which homology models, constructed based on the LmrR templates, scored better relative to the homology models constructed based on the other PadR‐s2 templates were considered as potential candidates with a larger likelihood to exhibit open‐pore structures similar to LmrR. These candidates were further assessed by visual analysis and multiple sequence alignment (MSA), ultimately resulting in a set of six PadR candidates for further experimental testing (Figures 3a,b and S1). The candidates, LCf‐, LB1‐, Bh‐, LB2‐, LSm‐, and PePadR, originate from a variety of organisms and have sequence identities of 40–50% compared to LmrR, and 40–65% between each other. Whereas conserved PadR motifs can be observed from the MSA (Figure 3b), it can also be seen that the candidates feature distinct differences, especially in the α4 helices that make up a large portion of the dimeric interfaces of these proteins, potentially yielding different active site microenvironments than LmrR.

### 2.2 Production and Characterization of PadR Candidates

Codon‐optimized genes for the six PadR candidates were ordered in pET17b vectors, including a C‐terminal StrepTag for affinity purification. Additionally, mutations known to decrease DNA‐binding of LmrR (K55D and K59Q) [48] were also introduced when positively charged residues were present at the homologous positions of the new PadR proteins (residues 56 and 60 in the MSA in Figure 3b). Proteins were then recombinantly produced in *E. coli*, using similar expression conditions as used for LmrR. Remarkably, all candidates could be purified as soluble proteins and with acceptable‐to‐good yields (20–150 mg/L). Purification of the target proteins was followed by SDS‐PAGE (Figure S2), and their identities were confirmed by LC‐MS (Figure S3). Size‐exclusion chromatography was performed to assess the oligomeric states of the proteins and showed that all candidates form dimeric structures, analogous to LmrR (Figure S3). Moreover, CD spectra indicated that all proteins exhibit α‐helical dominant secondary structures (Figure S4). Together, these experiments strongly suggest that the six candidates indeed belong to the PadR‐s2 protein family. Interestingly, as evaluated by thermofluor thermal shift assay, LCf and Bh exhibited significantly higher thermostabilities than the other four candidates (Table S1) [49]. It must be noted that a reliable apparent melting temperature (*T*_m‐app_) could not be obtained for LmrR using this method. LmrR likely binds the SYPRO orange dye used in this experiment within its open pore, leading to an abnormal melting curve (Figure S5) [50]. This behavior is not observed for the other PadR proteins, further indicating that their dimeric interfaces provide a different environment, without affinity for the dye. Thermostability was further assessed by 30‐min incubation of cell‐free extracts up to 70°C and subsequent SDS‐PAGE analysis of the remaining soluble fraction. Solely LCf and Bh survived heat treatment until at least 60°C and 70°C, respectively, becoming enriched at the same time (Figure S6).

**FIGURE 4:**
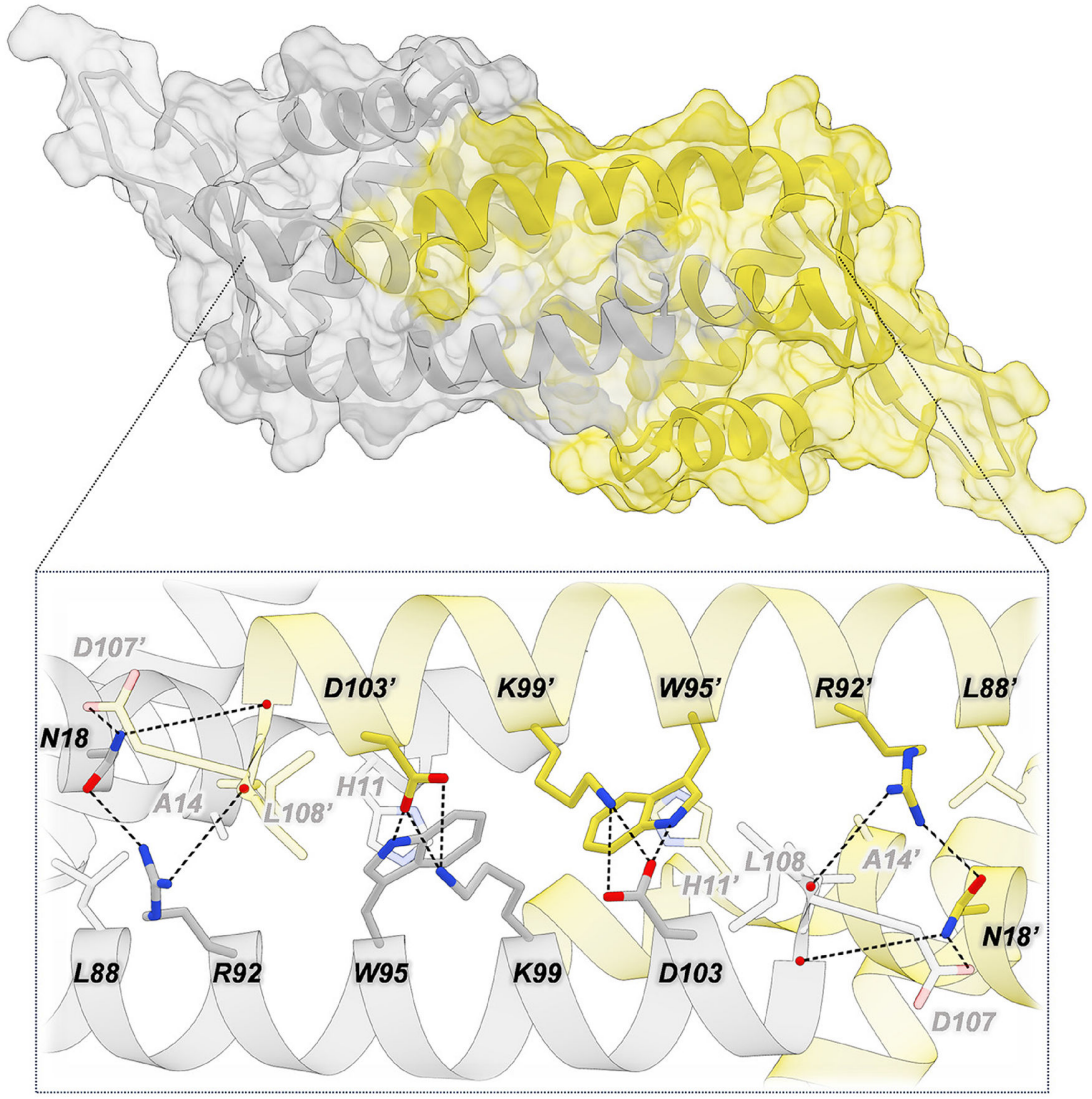
Surface representation of the crystal structure of BhPadR (PDB 9QBC), with a zoomed‐in view of residues located at the closed dimer interface. One of the dimer‐related monomers is depicted in yellow. Hydrogen bonds and electrostatic interactions are indicated by dotted black lines. Some of the observed hydrogen bonds of N18 and R92 are with backbone carbonyl oxygen atoms in the C‐terminal end of the opposing α4 helix, shown as red spheres in the displayed ribbon. Residues N18 and R92 are marked as green dots in the MSA (Figure 3b), whereas W95, K99, and D103 are marked with orange dots.

We were able to crystallize one of the new proteins, Bh, and solve its structure at a resolution of 2.05 Å (PDB 9QBC, Figure 4, Table S2). The crystal structure of Bh exhibits a closed dimeric interface, resembling most PadR‐s2 crystal structures and the AlphaFold predicted structure. Compared to LmrR, the two C‐terminal helices in Bh are shifted toward each other and interlock, positioning the side chains of the central tryptophans (W95 and W95’) side by side at the dimer interface instead of facing each other as observed in LmrR (Figures 2 and 4). The closed dimer interface in Bh is further stabilized by a network of electrostatic interactions and hydrogen bonds formed by residues spanning the interface. In particular, K99 interacts with D103’ in the opposing α4 helix, which in turn forms a hydrogen bond with W95 (Figure 4; residues depicted with an orange dot in the MSA in Figure 3b). The other PadR proteins harbor residues at equivalent positions that could support similar electrostatic or hydrogen bonding interactions as observed in Bh. In contrast, LmrR contains negatively charged residues at both positions, disfavoring their direct interaction due to electrostatic repulsion. Furthermore, R92 forms a hydrogen bond with a backbone carbonyl oxygen atom in the C‐terminal part of the opposing α4 helix and with N18 in the α1 helix (residues depicted with a green dot in the MSA in Figure 3b). Whereas N18 is conserved in both LmrR and the other PadR proteins, the residue corresponding to R92 is more variable; LmrR contains a phenylalanine at this position, which cannot participate in hydrogen bonding. Although alternative conformations may exist in solution, AlphaFold performed remarkably well in predicting the structure of Bh, as demonstrated by the near‐perfect alignment of the predicted model with the crystal structure (Figure S7). This supports the reliability of the predicted models for the other five PadR proteins and suggests that they may likewise favor a closed dimeric interface.

LmrR has previously been used as an artificial enzyme in a Lewis‐acid catalyzed FC‐alkylation reaction using Cu^II^(1,10‐phenanthroline)(NO_3_)_2_ (Cu^II^Phen) as a supramolecularly anchored cofactor inside the pore via stacking interactions with the two dimer related tryptophans W96 and W96’ (residue 97 in the MSA in Figure 3b) [51]. Whereas this reaction can be promoted by Cu^II^Phen itself, LmrR provides a chiral environment that allows enantioselective catalysis (Table 1, entries 1 and 2). As a straightforward comparison to LmrR, we tested our new PadR proteins in this FC‐alkylation reaction (Table 1, entries 3–8). While reactions in which LB1, Bh, LSm, and Pe were used as scaffolds did not display significant activity, a modest yield was obtained when using LCf or LB2. Controls without Cu^II^Phen catalyst did not show any significant activity (Table S3). Unfortunately, little to no enantioselectivity was observed when using LCf or LB2. An interesting observation, though, is the fact that reactions performed with PadR proteins have significantly lower yields than free Cu^II^Phen, especially in the case of LB1, Bh, LSm, and Pe. Among others, a possible explanation could be that the scaffolds can bind Cu^II^Phen, but in a fashion that does not promote catalysis. Relating these results to the obtained crystal structure (Figure 4), it could be that Bh can accommodate Cu^II^Phen through potential stacking interactions with the central tryptophans. However, binding may occur in a conformation that does not expose the catalytic Cu^II^Phen to the substrates, and/or the limited space of the closed dimeric interface of Bh restricts access and binding of substrates, preventing catalysis. In the case of LCf and LB2, the affinity for Cu^II^Phen may be lower, resulting only in partial inhibition of the racemic background reaction.

**TABLE 1:** Results for the Cu^II^phen‐promoted FC‐alkylation reaction between 1 and 2 using different PadR proteins.

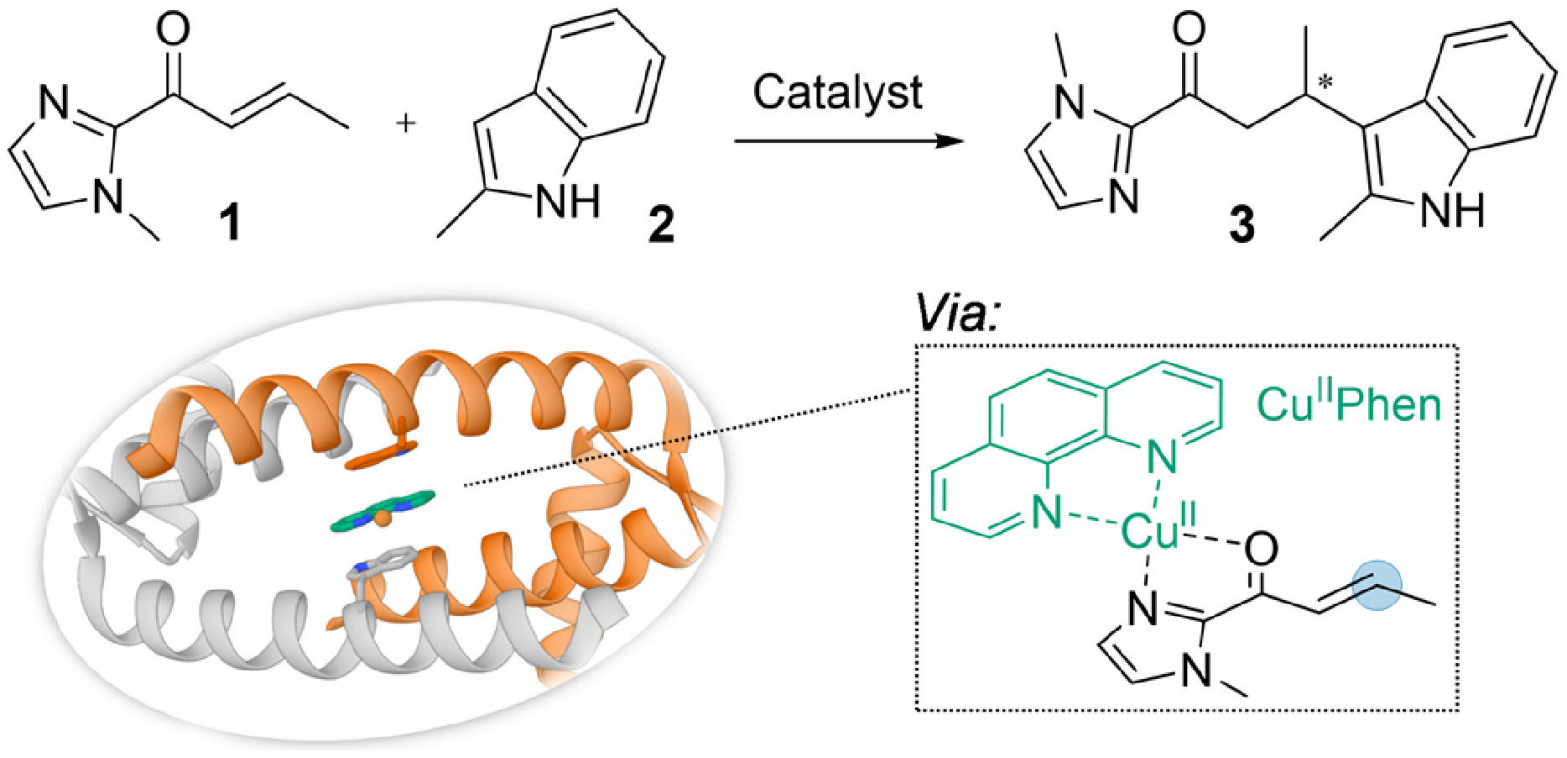			
Entry	Catalyst	Yield, %	ee, %
1	Cu^II^Phen	59 ± 3	1 ± 1
2	**LmrR⊃**‍Cu^II^Phen	54 ± 3	94 ± 1
3	**LCf⊃**Cu^II^Phen	24 ± 1	2 ± 0
4	**LB1⊃**Cu^II^Phen	2 ± 0	0 ± 5
5	**Bh⊃**Cu^II^Phen	4 ± 0	1 ± 1
6	**LB2⊃**Cu^II^Phen	31 ± 3	3 ± 1
7	**LSm⊃**Cu^II^Phen	3 ± 0	1 ± 1
8^a^	**Pe⊃**Cu^II^Phen	5 ± 1	7 ± 1

The depicted protein is the crystal structure of LmrR, showing supramolecular bound Cu^II^Phen (PDB 6R1L) [45]. Analytical yield and enantiomeric excess (ee) of product **3** were determined by high performance liquid chromatography. Entries are based on at least three experiments, using two independently produced batches of protein. Errors are the standard deviation of the results. Reaction conditions: PadR protein (60 µM dimer), Cu^II^Phen (45 µM), **1** (1 mM), **2** (1 mM) in 3‐(N‐morpholino)propanesulfonic acid (MOPS) buffer (20 mM, pH 7.0) containing NaCl (150 mM) and DMSO (5% v/v) in a total volume of 300 µL, continuously inverted for 48 h at 4°C.

a Precipitation was observed when using Pe under these conditions.

### 2.3 Genetic Incorporation of NcAAs and Iminium Catalysis

**FIGURE 5:**
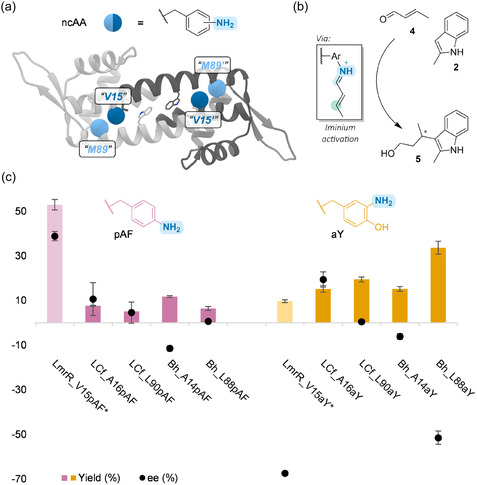
(a) Location of the positions analogous to LmrR “V15” and “M89” depicted in the crystal structure of BhPadR. (b) FC‐alkylation of **2** with **4** via iminium activation. (c) Analytical yield and ee (determined by supercritical fluid chromatography) of the FC‐alkylation reaction between **2** and **4** for the different PadR variants harboring either pAF or aY as catalytic residue at the analogous LmrR “V15” or “M89” positions. Reaction conditions: LmrR_V15pAF (20 µM dimer) or LmrR_V15aY / PadR variants (60 µM dimer), **2** (1 mM), and **4** (5 mM) in phosphate buffer (50 mM, pH 6.5) for variants harboring pAF or 2‐(N‐morpholino)ethanesulfonic acid (MES) buffer (20 mM, pH 5.5) for variants harboring aY, containing NaCl (150 mM) and DMF (8% v/v) in a total volume of 300 µL, incubated in a thermomixer (750 rpm) for 16 h at 8°C, followed by reduction with NaBH_4_, to yield the corresponding alcohol product **5**. Entries are based on at least five experiments, using two or more independently produced batches of protein. Errors are the standard deviation of the results. ee is assigned relative to the enantiomer obtained with LmrR_V15pAF, with negative values representing the opposite enantiomer. *Data from Brouwer et al. [28].

The site‐specific incorporation of ncAAs featuring catalytic functionalities into protein scaffolds via stop codon suppression has proven to be an appealing method to expand the catalytic repertoire of enzymes [18, 19]. Whereas the promiscuous binding pore of LmrR has served as good starting point for the creation of rudimentary artificial enzymes, such a predefined binding pocket might not be necessary in all cases. As long as a basal amount of desired reactivity can be found, directed evolution can be applied in an effort to increase the artificial enzyme's performance. Therefore, even though the new PadR proteins appear to lack binding pores, we envisioned it would still be interesting to assess their applicability as scaffolds by trying to incorporate ncAAs. We investigated the incorporation of ncAAs at the equivalent positions of V15 and M89 in LmrR (residues 16 and 90 in the MSA in Figure 3b). These positions have previously been used to create LmrR‐based artificial enzymes featuring an ncAA and are both positioned at the dimeric interface, with "V15" being more inside the pore and "M89" being closer to the surface and on the side of the dimeric interface (Figure 5a) [26, 27, 52]. The homodimeric nature of PadR‐s2 proteins ultimately leads to the introduction of two ncAAs per assembly.

From SDS‐PAGE analysis of small‐scale expressions, a trend became evident that ncAA incorporation only leads to soluble proteins in the case of LCf and Bh (Figure S8). Larger‐scale expression and purification were subsequently performed for these two proteins, LCf and Bh, incorporating either p‐azidophenylalanine (pAzF) or 3‐aminotyrosine (aY) at the LmrR equivalent “V15” or “M89” positions (Figure S9). Following purification, pAzF was post‐translationally reduced with tris‐(2‐carboxyethyl)phosphine to obtain p‐aminophenylalanine (pAF) for later application in catalysis. Correct incorporation of pAF or aY was confirmed by LC‐MS (Figure S10). NcAA incorporation was found to lower the *T*_m‐app_ of the proteins to various degrees, depending on the ncAA and the position of incorporation (Table S1). Incorporation of either pAF or aY at position 90 in LCf, for example, severely lowered the *T*_m‐app_ by 15–16°C. This demonstrates that incorporation of an ncAA can significantly affect the protein stability, and whereas the destabilizing effects are partly offset by the relatively high starting thermostabilities of the LCf and Bh parent proteins, this may not have been the case for LB1, LB2, LSm, and Pe.

pAF and aY have previously been employed as catalytic residue in LmrR, promoting enantioselective vinylogous FC‐alkylations via iminium activation (Figure 5b,c) [28, 53]. We decided to test the LCf_ and Bh_pAF/aY variants in the same reaction, and to our delight, the new designer enzymes displayed low to moderate levels of activity (Figure 5c). Moreover, varying enantiopreferences were observed, indicating that the proteins provide different chiral environments. For example, variants harboring aY at position "V15," LCf_A16aY and Bh_A14aY, favor opposite enantiomers. Controls without pAF or aY resulted in lower activities and enantioselectivities, suggesting the involvement of the ncAAs as catalytic residues (Table S4). Interestingly, variants harboring aY gave rise to higher yields than variants harboring pAF. This is in contrast with LmrR, in which pAF was more active. Of all new variants, Bh_L88aY shows the best results for this FC‐alkylation reaction, exhibiting significant enantioselectivity (52% ee) and a threefold higher yield than LmrR_V15aY. Had LmrR_V15aY not already been evolved further for this transformation [28], Bh_L88aY would have made a good alternative starting point for directed evolution. Although LCf and Bh were both predicted or observed to exhibit a closed dimeric interface, variants harboring ncAAs could still perform catalysis, suggesting the catalytic residues are accessible to substrates.

**FIGURE 6:**
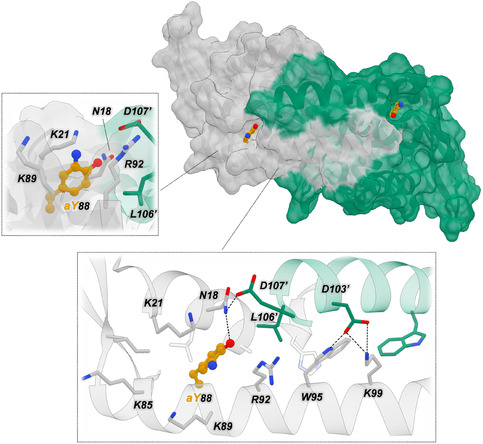
Surface representation of the crystal structure of Bh_L88aY (PDB 9QBD), showing two different zoomed‐in views of one of the genetically incorporated aYs. One of the dimer‐related monomers is depicted in green, and aY is depicted in orange. Hydrogen bonds or electrostatic interactions are depicted as dotted black lines.

We were able to solve the crystal structure of Bh_L88aY at a resolution of 2.10 Å (PDB 9QBD, Table S2). Incorporation of the aY residues had a minimal effect on the overall dimeric Bh structure, being positioned relatively close to the surface at either side of the closed dimeric interface (Figure 6). The aminophenol side chain of aY88 resides in a shallow crevice, and the hydroxyl group forms a hydrogen bond with N18, positioning the side chain of aY in such a way that the primary amine functionality is directed toward the surface of the protein (Figures 6 and S11). Directly surrounding aY88, three positively charged amino acids, K21, K89, and R92, are observed (Figure 6). Together with other residues in the immediate surroundings of aY88, they prevent the catalytic amine from being fully exposed on the surface and likely contribute to enantioselectivity by modulating substrate access. These observations are in contrast with LmrR‐based Friedel–Crafts alkylases, for which the reaction is believed to occur at the center of the dimeric interface, involving the two central tryptophans and an acidic residue (D100) [28, 54]. This suggests that an open pore, like observed in LmrR, is not a strict requirement for constructing a rudimentary enzyme based on a PadR scaffold. Moreover, the strikingly different immediate surroundings of the catalytic ncAA in Bh_L88aY compared to previous LmrR‐based FC‐alkylases may allow selectivities and/or reactivities that were not attainable before.

## 3 Conclusion

In this study, we aimed to enlarge the scope of protein scaffolds that can be used in the creation of artificial enzymes and performed a bioinformatics study to find homologs of the successful PadR scaffold LmrR. Six previously uncharacterized PadR proteins were recombinantly produced, characterized, and evaluated in FC‐alkylation catalysis. In contrast with homology models of the new PadR proteins, AlphaFold predictions resulted in structures that exhibit closed dimeric interfaces. In the case of BhPadR, this was confirmed by the experimentally determined X‐ray crystal structure. In our bioinformatics study, we were not able to find PadR proteins with an open‐pore structure, highlighting the unique characteristics of LmrR, the origin of which is currently not understood. The closed dimeric interfaces of the new PadR proteins were found not to be suitable for the FC‐alkylation reaction promoted by supramolecularly anchored Cu^II^Phen. Two of the proteins, however, LCf and Bh, were found to be amenable to the incorporation of ncAAs and could subsequently be used as artificial enzymes in the iminium‐promoted FC‐alkylation using pAF or aY as catalytic residue. Incorporation of the ncAAs at different positions in the two PadR proteins led to varying reactivities and selectivities, demonstrating that the scaffolds provide different chemical environments for FC‐catalysis. The experimentally determined X‐ray crystal structure of one of the more active constructs, Bh_L88aY, showed a closed‐pore structure with aY88 positioned in a shallow crevice close to the surface of the protein. The catalytic site of Bh_L88aY was found to be distinctly different than previous artificial FC‐alkylases based on LmrR. Overall, these results demonstrate that the open‐pore structure of LmrR is not a prerequisite per se for the design of artificial FC‐alkyases and that, even though they exhibit closed dimeric interfaces, LCf and Bh can be used as scaffolds for artificial enzymes. Our findings thus demonstrate that closed PadR‐s2 proteins should not a priori be discounted in the design of artificial enzymes, and we provide the field with two new PadR scaffolds for future designs.

## Supporting Information

Additional supporting information can be found online in the Supporting Information section.

## Funding

This work was supported by the Nederlandse Organisatie voor Wetenschappelijk Onderzoek (OCENW.KLEIN.143), the European Research Council (885396), and the European Synchrotron Radiation Facility (MX‐2649).

## Conflicts of Interest

The authors declare no conflicts of interest.

## Supporting information

Supplementary Material

## Data Availability

Crystallographic data have been deposited in the Protein Data Bank (PDB) under accession codes 9QBC (BhPadR) and 9QBD (Bh_L88aY). Raw diffraction images collected at the ESRF for Bh_L88aY are available via the data DOI: 10.15151/ESRF‐ES‐1909180024.
