# Supplementary material for: Exploring PadR Proteins for Artificial Enzyme Design
Source: Chembiochem. 2026 Apr 28;27(8):e70308. doi: 10.1002/cbic.70308 (PMC13122737; doi:10.1002/cbic.70308)
Supplement: Supplementary file 1 — Supplementary Material [file CBIC-27-e70308-s001.pdf]

Supporting Information

Exploring PadR Proteins for Artificial Enzyme Design

Bart Brouwer,[a] Andy-Mark W.H. Thunnissen,[b] Henriette J. Rozeboom,[b] and Gerard Roelfes[a]*

[a]Stratingh Institute for Chemistry, University of Groningen, Nijenborgh 3, 9747 AG Groningen, The Netherlands. [b]Groningen Biomolecular Sciences and Biotechnology Institute, University of Groningen, Nijenborgh 3, 9747 AG Groningen, The Netherlands.

*Corresponding author e-mail address: j.g.roelfes@rug.nl.

Table of Contents

1. Mining PadR Proteins S2
2. Structure Prediction and Homology Modeling S3
3. Supporting Figures S5
4. Supporting Tables S14
5. General Information S17
6. Cloning of PadR constructs S18
7. Protein Production and Purification S20
8. Size Exclusion Chromatography S21
9. Thermofluor Assay S21
10. Protein Crystallography S22
11. Protein Mass Spectrometry S22
12. Catalysis and Workup Procedures S23
13. Synthetic Procedures and Characterization S24
14. Calibration Curves and HPLC Chromatograms S24
15. References S27

1. Mining PadR Proteins

The sequence of LmrR (Uniprot code A2RI36, PDB 3F8B) was used as query for a protein BLAST search (https://blast.ncbi.nlm.nih.gov/Blast.cgi)[1] on the 1st of april-2020, using default settings with a maximum of 5000 target sequences. It is interesting to note that there were little to no sequences found with an identity percentage between 60-90% compared to LmrR, demonstrating the uniqueness of this protein. The resulting sequences were filtered, keeping only sequences within a range of 25-100% identity and E-values between 2e-^{78} and 8e-^{10}. To this database, eleven known PadR-s2 sequences were added (PDB 3F8B, 1XMA, 3HHH, 3L7W, 4EJO, 4ESB, 4ESF, 5DYM, 5H20, 5ZQH, 6ABQ) for future reference. The MAFFT webserver (version 7, https://mafft.cbrc.jp/alignment/ server/index.html)[2] was used to limit the sequence length to 99-130 amino acids, resulting in a database of 3806 putative PadR-s2 proteins. MAFFT was then used to create a MSA and to construct a phylogenetic tree (Average linkage UPGMA, default settings). In parallel, the same database was used to create a MSA and phylogenetic tree (NJ) with the ClustalOmega webserver (https://www.ebi.ac.uk/jdispatcher/msa/clustalo) using default settings.[3] The eleven known PadR-s2 sequences were localized in these trees and clades close to LmrR were selected and extracted, while omitting clades harboring the added known closed-pore PadR-s2 sequences (see workflow below, note that for clarity whole branches are colored in the phylogenetic trees to display LmrR or the other closed-pore PadR sequences). It was observed that the added PadR-s2 sequences of PDBs 1XMA and 5DYM were generally closer to LmrR in the phylogenetic tree than the other added PadR-s2 PDBs. The thus obtained sequences were combined and redundancy removed using the CD-HIT functionality within the MAFFT webserver. As the number of sequences reduced, more stringent settings could be used create new phylogenetic trees with improved accuracy. The known PadR-s2 sequences were again added to this database (1249 sequences) and subsequently used to create three new MSAs and phylogenetic trees (ClustalOmega (NJ), MAFFT (NJ;conserved sites 30 AAs) and MAFFT (NJ;all of gap-free sites 50 AAs)). Once more, the added known PadR-s2 sequences were localized, and clades close to LmrR extracted, combined and redundancy taken out. The resulting sequences were then further filtered using CD-HIT to exclude sequences with 98% similarity, resulting in the final database containing 112 putative PadR sequences, for which a MSA and phylogenetic tree (ClustalOmega (NJ)) was constructed after adding the sequence of LmrR (see workflow below). Jalview and the built-in ClustalO webservice (with defaults) was used for multiple sequence alignments.[4] Archearepotyx (https://www.phylosoft.org/archaeopteryx/) and iToL[5] were used for visualizing phylogenetic trees.

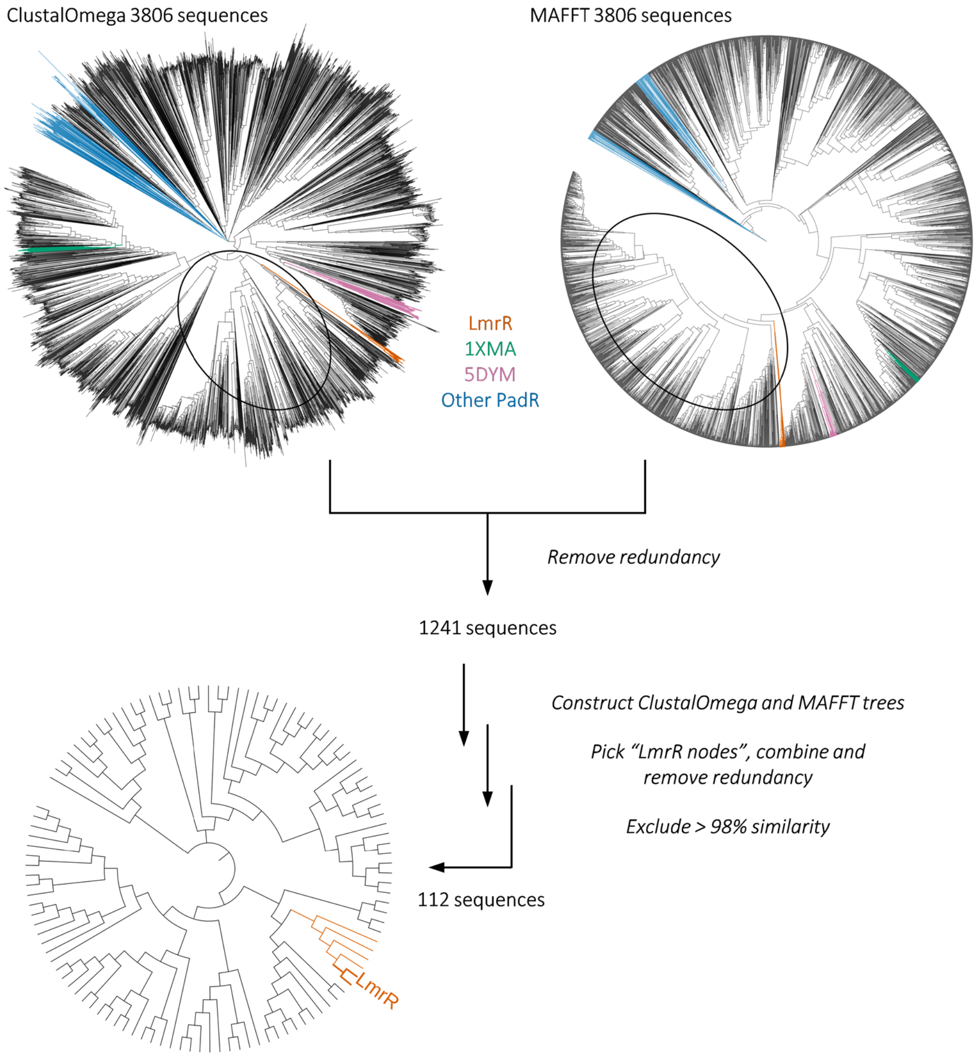

2. Structure Prediction and Homology Modeling

AphaFold structure predictions of the 112 putative PadR sequences were performed using a local installation of AlphaFold2.1.1-fosscuda-2020b on a high-performance cluster with multimer settings and a *max_template_date* of 2022-03-31.[6,7] All PadR-s2 sequences modeled with AlphaFold, including the six experimentally produced candidates, resulted in predicted structures resembling the more closed-pore PadR protein crystal structures (**Figure S1**). For a large portion of the 112 putative PadR sequences, homology models (HMs) were constructed and refined with YASARA Structure (version 19.12.14) using the YASARA2 force field.[8] An overview of the workflow is shown below. For each sequence that was modeled, fourteen HMs based on different templates were created using an adapted YASARA macro. As templates, four LmrR crystal structures (PDBs 3F8B, 3F8F, 6I8N and 6VWE) and ten PadR-s2 crystal structures (1XMA, 3HHH, 3L7W, 4EJO, 4ESB, 4ESF, 5DYM, 5H20, 5ZQH and 6ABQ) were used. The following adaptions were made to the default YASARA *hm_build* macro used for homology modeling:

- *templates=14*
- *termextension=30*
- *Templates (templates),SameSeq=2*
- *DelTemplateRes !Protein*
- *TemplateList 3F8B,3F8F,6I8N,4ESB,4ESF,5H20,1XMA,5DYM,3L7W,6ABQ,3HHH,5ZQH,4EJO*
  Note: the template model for 6VWE was manually provided and named target_T001 as the respective structure found from the PDB by YASARA did not result in dimeric homology models.
- *LoopLenMax 20*

HMs were scored based on their ModelQuality (MQ), using knowledge-based potentials (0.145*'Dihedrals', 0.390*'Packing1D' and 0.465*'Packing3D) integrated in YASARA.[8] For the five top ranked HMs, and at least the highest scored LmrR-based HM, the three highest scored other PadRs-s2-based HMs, and the 1XMA and 5DYM based HMs, short 0.5 ns molecular dynamics (MD) simulations were performed to refine the HMs. The YASARA *md_refine* macro with default settings was used for the MD simulations. The macro was slightly adapted to also calculate and report MQ scores of 25 snapshots taken over the course of the MD simulation. The highest scoring snapshot of all MD simulations performed for the different HMs for a given sequence were subsequently compared. For each sequence, the ∆MQ score (defined as the MQ score of the snapshot of the highest scored LmrR-based model subtracted by the MQ score of the snapshot of the highest scored other PadRs2-based model) was calculated and considered as a measure to assess the likelihood that a given sequence could exhibit an LmrR-like structure.

Sequence
WP_107926394

Homology Modeling

Template HM	MQ score
3F8B	0.506
3F8F	0.333
5DYM	0.206
1XMA	0.195
6I8N	0.185
4EJO	0.007
6VWE	-0.003
5H20	-0.005
3HHH	-0.025
4ESF	-0.089
5ZQH	-0.091
4ESB	-0.149
3L7W	-0.185
6ABQ	-0.347

0.5 ns MD of top 5 HMs

At least:
best LmrR HM
best 3 PadR HMs
1XMA/5DYM HMs

Template HM	MQ score best snapshot MD	Best model for LmrR/PadR
**3F8B**	**0.752**	Best LmrR
3F8F	0.449	
5DYM	0.163	
**1XMA**	**0.441**	Best other PadR
6I8N	0.501	
4EJO	0.104	

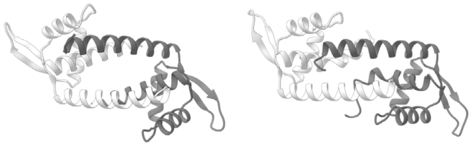

3F8B model (**0.752**) - 1XMA model (**0.441**)

∆MQ for WP_107926394 = **0.311**

To validate the use of the ∆MQ score, controls were performed by applying the above-mentioned workflow on sequences of some of the template crystal structures. The ∆MQ score was indeed found to be positive when modeling LmrR, and low to negative when modeling other PadR-s2 template sequences (see the table on the next page). Next to that, several sequences from nodes far away from LmrR in the phylogenetic trees, that were discarded during refinement of the PadR sequence database (*), were tested and resulted in low to negative ∆MQ scores as well. Based on these controls, a threshold for desirable ∆MQ score was set at a value of 0.2 and above. This workflow was then applied to in total 52 sequences, modeling at least one sequence of every larger clade found in the phylogenetic tree of the 112 putative PadR sequences. When a ∆MQ score >0.3 was obtained, also other sequences from that clade were modeled. Of the 52 modeled sequences, 15 sequences were obtained with ∆MQ scores >0,3 and 13 sequences with a ∆MQ score between

0.2-0.3. (see tree below). The models and MSAs of these 28 proteins were visually analyzed to make a selection of PadR candidates for experimental testing. Selections were based on the absence or presence of features that were observed when comparing LmrR with closed-pore PadR-s2 crystal structures. Among others, such features included: The presence of multiple residues in the opposite α4 helices with similarly charged side chains facing the dimeric interface (as observed in LmrR), potentially favoring an open-pore by electrostatic repulsion; Hydrophobic or stacking interactions of the central tryptophans with other residues in the dimeric interface, potentially favoring a closed-pore; Interactions between residues in the a1 and a4' helices, potentially favoring an open-pore; The presence of H-bonding or electrostatic interactions between residues in the opposite α4 helices (as observed in several closed-pore PadR-s2 crystal structures), potentially favoring a closed-pore. For example, if a homology model based on a closed-pore PadR-s2 crystal template structure clearly showed interactions between residues lining the α4 helices of the dimeric interface, this would be interpreted as a potential factor favoring a closed-pore structure for that sequence. On the other hand, if such interactions were not observed, this would be interpreted as a factor potentially favoring an open structure for that sequence, and thus selection as a candidate for experimental testing. Next to the analysis of above-mentioned features, candidates were also chosen to feature diverse overall charges, polarity and residues lining the dimeric interface in an effort to provide PadR proteins with varying microenvironments. A selection of six PadR candidates was made for experimental testing: **LCf**PadR (Genbank PCS01472); **LB1**PadR (Genbank WP_137635460); **Bh**PadR (Genbank WP_107926394); **LB2**PadR (Genbank WP_127848481); **LSm**PadR (Genbank PAK80441); **Pe**PadR (Genbank WP_057805030).

Sequence	ΔMQ
3F8B	0.786
1XMA	-0.079
5DYM	0.121
3HHH	-0.503
3L7W	-0.505
4EJO	-0.583
4ESB	-0.820
5ZQH	-0.493
WP_036096607*	-0.106
WP_138196879*	0.128
WP_091739993*	-0.294
PCS01472(**LCf**)	0.503
WP_137635460 (**LB1**)	0.346
WP_107926394 (**Bh**)	0.311
WP_127848481 (**LB2**)	0.440
PAK80441(**LSm**)	0.220
WP_057805030 (**Pe**)	0.208

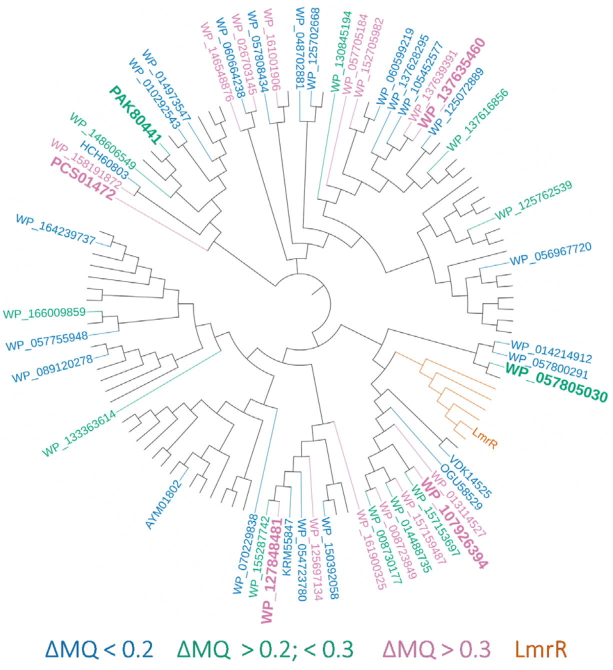

3. Supporting Figures

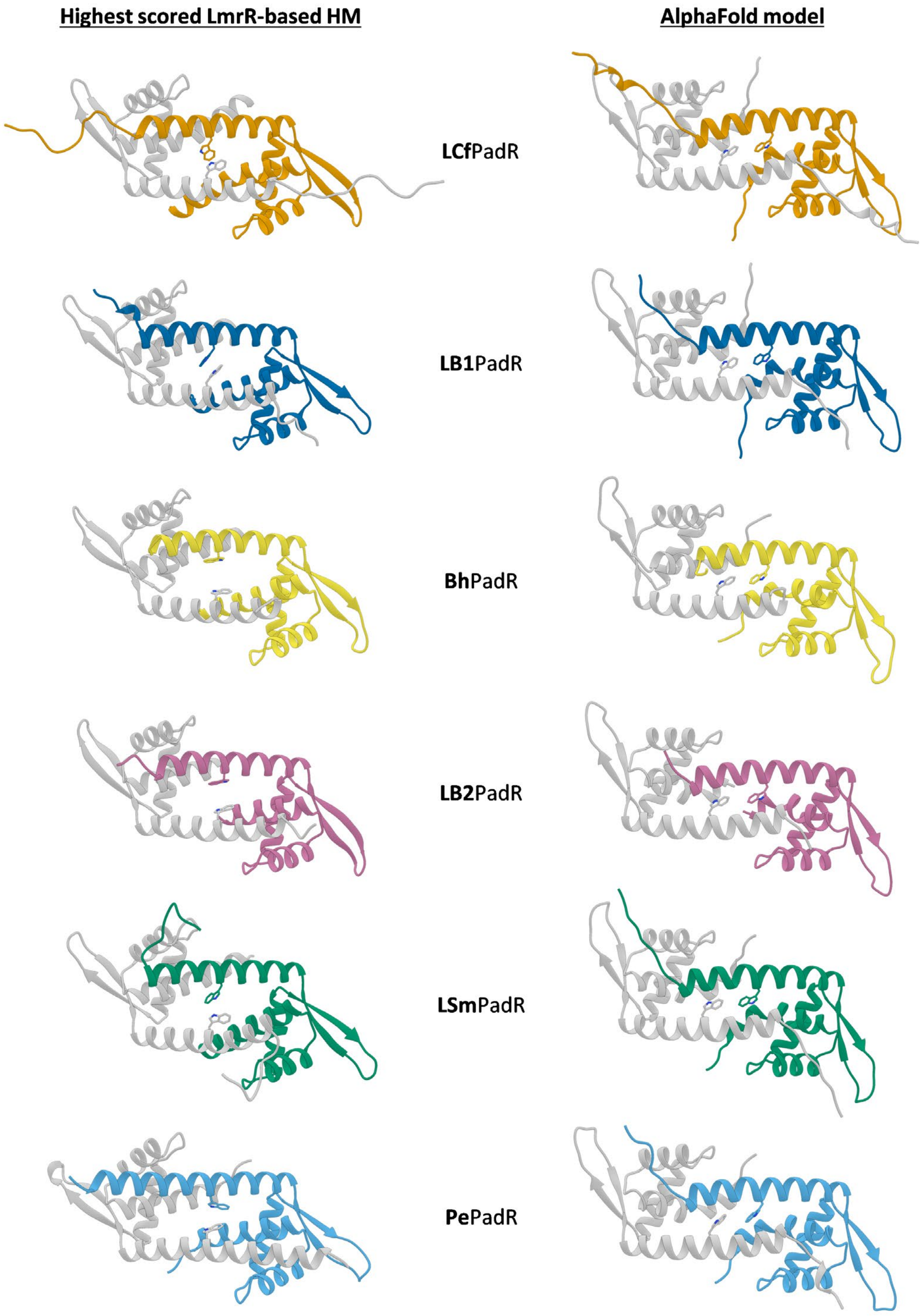

Figure S1. Highest scored LmrR-based homology models (left) and AlphaFold predictions (right) of the six PadR candidates. Conserved central tryptophans (W96 and W96’ in LmrR) are displayed as sticks in each model.

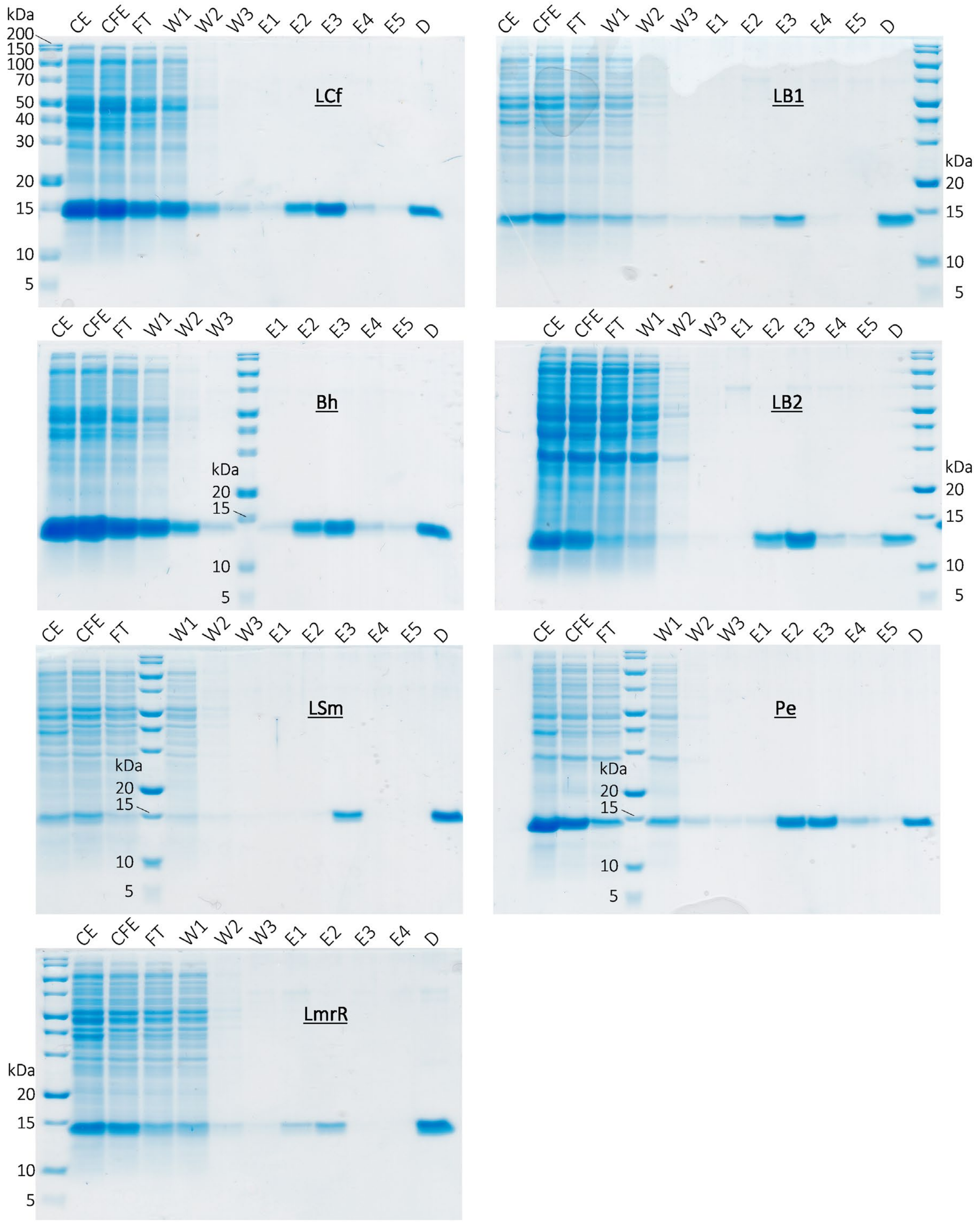

Figure S2. StrepTag purification of PadR proteins followed by SDS-PAGE (12% polyacrylamide, Tris-Tricine). CE = cell extract, CFE = cell-free extract, FT = flow-through, W = wash, E = elution and D = dialyzed purified protein. Samples of ≈ 0.1 OD_{600} units were loaded per well. For dialyzed pure protein, 10 µL of a 5 µM sample (dimer concentration) was loaded. Ladder = Thermo Scientific PageRuler unstained broad-range protein ladder. Expected masses of target PadR proteins range from 12.9-15.5 kDa (monomer).

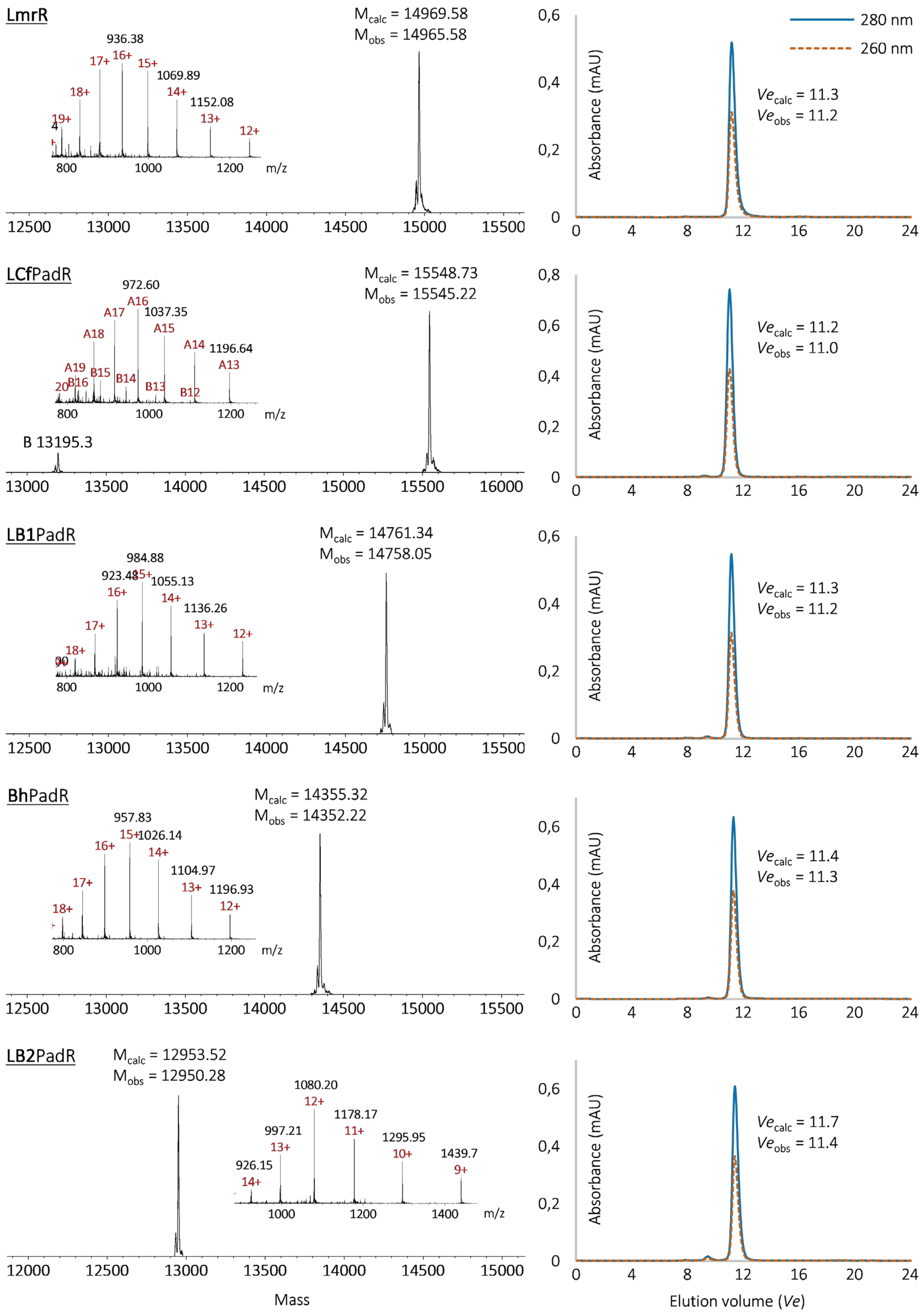
LmrR
Mcalc = 14969.58
Mobs = 14965.58
936.38
17+ 16+ 15+
1069.89
18+ 14+
1152.08
19+ 13+
12+
800 1000 1200 m/z
12500 13000 13500 14000 14500 15000 15500
280 nm
260 nm
Vecalc = 11.3
Veobs = 11.2
LCfPadR
Mcalc = 15548.73
Mobs = 15545.22
972.60
A16
A17 1037.35
A18 A15
A14 1196.64
A13
A19 B15 B14
20 B16 B13 B12
800 1000 1200 m/z
B 13195.3
13000 13500 14000 14500 15000 15500 16000
Vecalc = 11.2
Veobs = 11.0
LB1PadR
Mcalc = 14761.34
Mobs = 14758.05
984.88
923.48 15+ 1055.13
16+ 14+
1136.26
17+ 13+
12+
18+
800 1000 1200 m/z
12500 13000 13500 14000 14500 15000 15500
Vecalc = 11.3
Veobs = 11.2
BhPadR
Mcalc = 14355.32
Mobs = 14352.22
957.83
15+ 1026.14
16+ 14+
1104.97
17+ 13+ 1196.93
12+
18+
800 1000 1200 m/z
12500 13000 13500 14000 14500 15000 15500
Vecalc = 11.4
Veobs = 11.3
LB2PadR
Mcalc = 12953.52
Mobs = 12950.28
1080.20
12+
1178.17
997.21 11+
13+ 1295.95
10+ 1439.7
926.15 9+
14+
1000 1200 1400 m/z
12000 12500 13000 13500 14000 14500 15000
Mass
Vecalc = 11.7
Veobs = 11.4
Absorbance (mAU)
0 0,2 0,4 0,6 0,8
0 4 8 12 16 20 24
Elution volume (Ve)

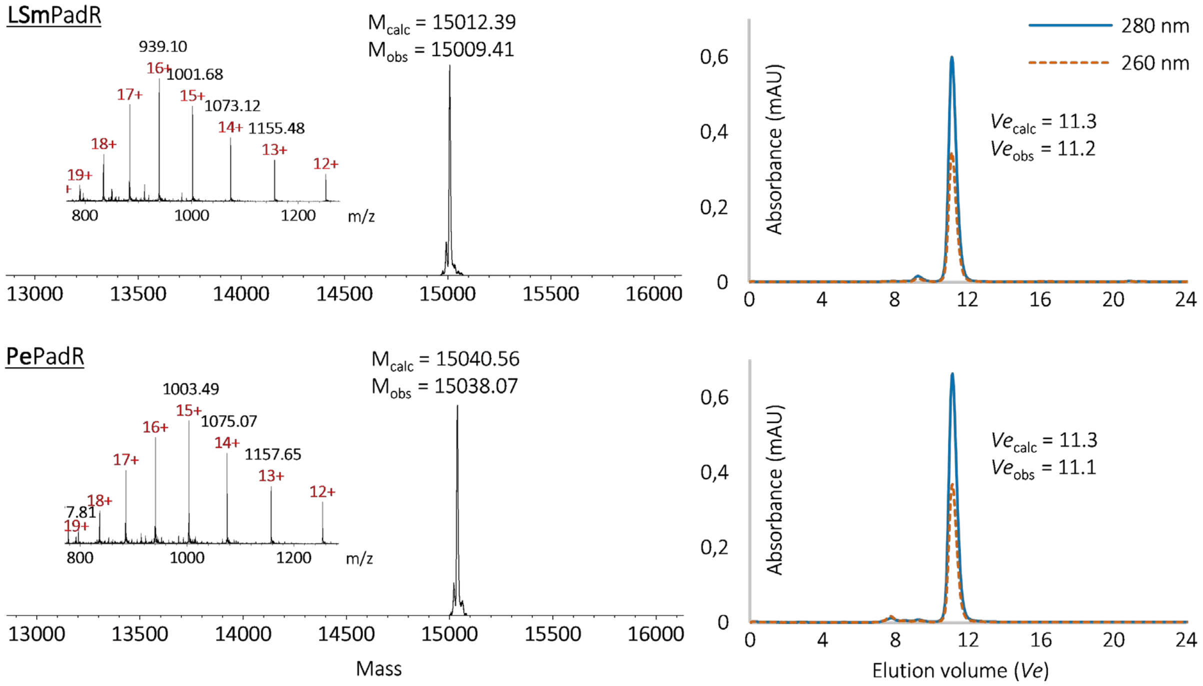

Figure S3. MS (ESI-QTOF) analysis, showing the obtained mass spectra and their deconvolution (left), and analytical size-exclusion chromatography (right) of purified PadR proteins. For experimental details, see SI sections 8 and 11. For LmrR and LCf, the N-terminal methionine was found to be cleaved off during protein production. For LCf, minor species corresponding to N- or C-terminal cleavage of larger peptides was occasionally observed.

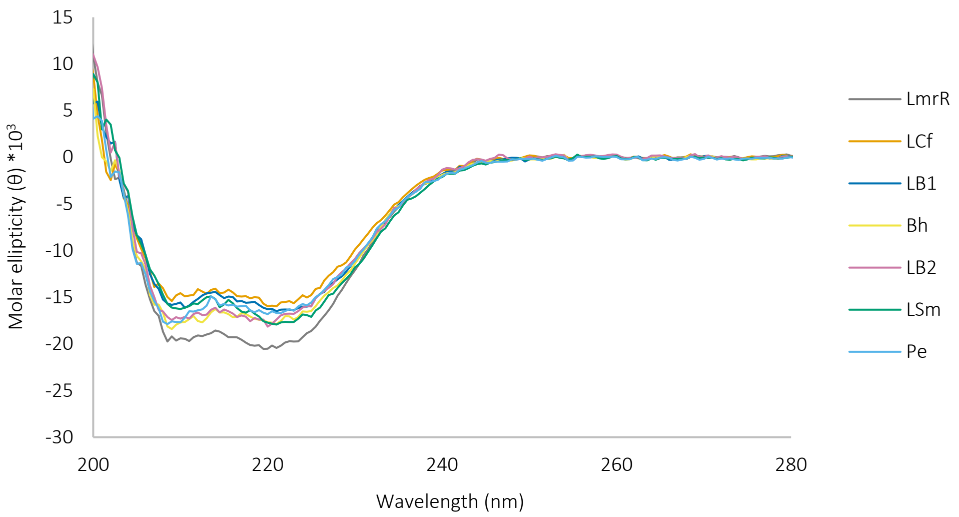

Figure S4. CD-spectra of purified PadR proteins (4 μM in 20 mM MOPS, 150 mM NaCl, pH 7). Measurements were recorded on a Jasco J815 CD spectropolarimeter using quartz cuvettes with a pathlength of 1 mm. Spectra were recorded between 190-300 nm with a scanning speed of 100 nm/min, a bandwidth of 2 nm, a digital integration time of 0.5 nm and 3 accumulations. The baseline was recorded with a sample containing only buffer.

a - MOPS pH 7.0:

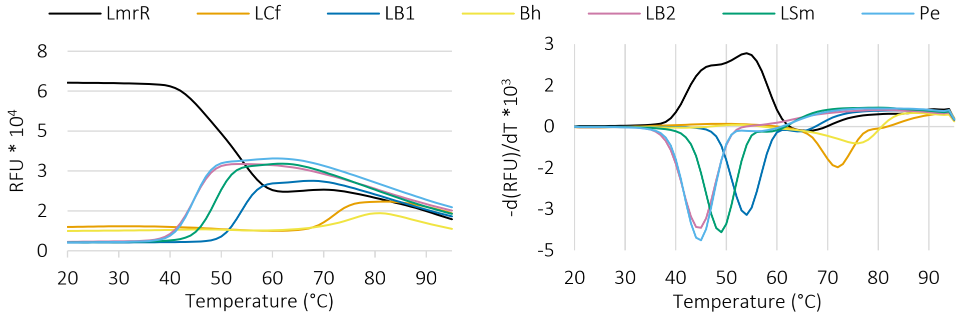

b - PBS pH 6.5:

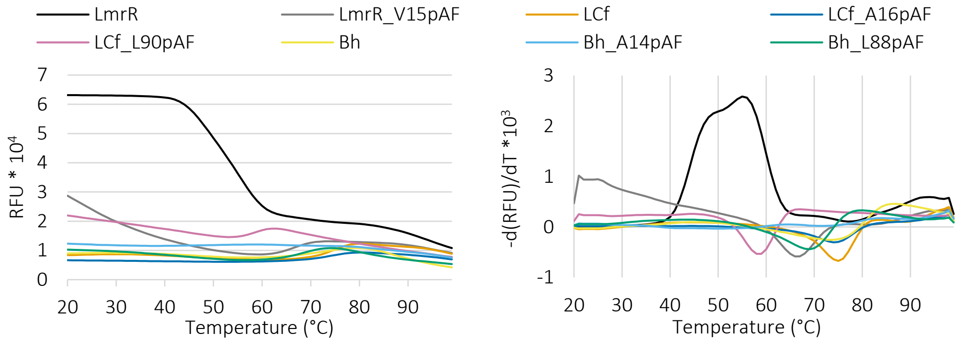

c - MES pH 5.5:

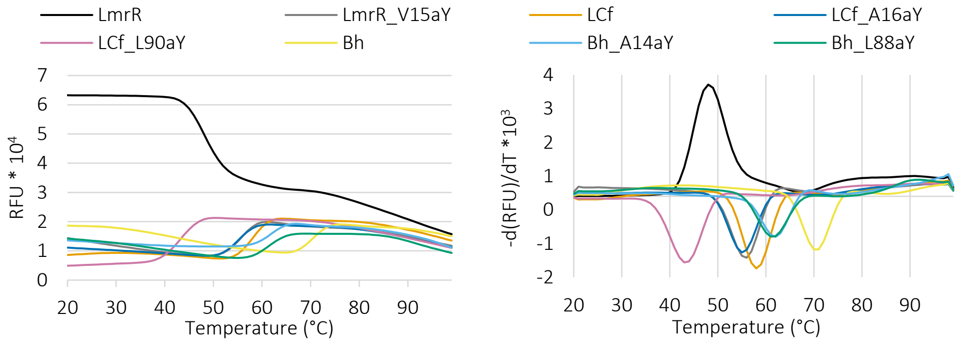

Figure S5. Relative fluorescence units (RFU) (left) and dRFU/dT (right) curves of thermofluor experiments. (a) Experiments performed using MOPS pH 7.0 buffer (20 mM MOPS, 150 mM NaCl, pH 7.0). (b) Experiments performed using PBS pH 6.5 buffer (50 mM Na_2HPO_4, 150 mM NaCl, pH 6.5). (c) Experiments performed using MES pH 5.5 buffer (20 mM MES, 150 mM NaCl, pH 5.5). For clarity, only one of the technical replicates per protein is shown. See **Table S1** for determined $T_{m\text{-}app}$ values, and SI section 9 for more details. Measurements of LmrR (without ncAA incorporated) exhibit high starting fluorescence, which may be due to binding of the SYPRO orange dye in the pocket of LmrR before denaturation.[9] This resulted in abnormal melting peaks in the dRFU/dT plots, and therefore, the $T_{m\text{-}app}$ of LmrR samples were not determined. A $T_{m\text{-}app}$ of 65 °C for LmrR is reported in the literature.[17] Interestingly, in contrast to LmrR, LmrR_V15pAF and V15aY show much lower starting fluorescence, making it possible to reliably determine the $T_{m\text{-}app}$ values for these variants. It could be that introduction of these ncAAs into LmrR at position V15 affect the pocket environment in such a way that causes reduced binding of the SYPRO orange dye, thus significantly lowering starting fluorescence compared to LmrR without ncAA. In PBS pH 6.5, LCf_L90pAF shows a relatively higher starting fluorescence than other LCf and Bh variants. It could be that introduction of pAF at position L90 in LCf affects the protein structure in such a way that hydrophobic parts become more exposed, leading to increased interaction with the SYPRO orange dye and thus a higher starting fluorescence.

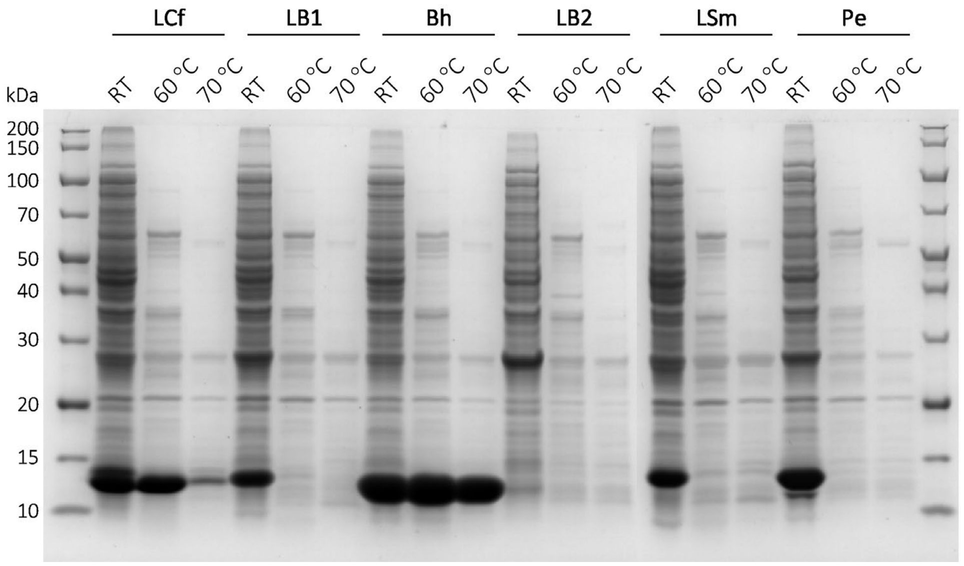

Figure S6. Heat treatment of cell-free extracts (CFEs) of cultures expressing PadR variants. CFEs were incubated at room temperature (RT), 60 °C or 70 °C for 30 min, precipitant was subsequently spun down and the remaining supernatant analyzed by SDS-PAGE (12% polyacrylamide, Bis-Tris). Note: LB2 did not express well in this particular batch. Ladder = Thermo Scientific PageRuler unstained broad-range protein ladder. Expected masses of target PadR proteins range from 12.9-15.5 kDa (monomer).

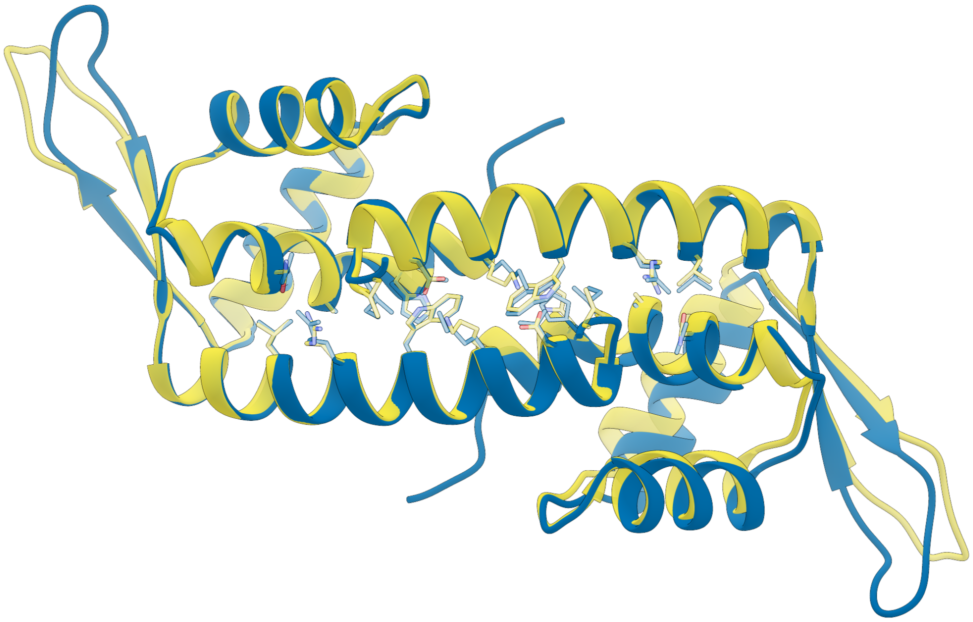

Figure S7. Comparison of the crystal structure of BhPadR (PDB 9QBC, yellow) and the AlphaFold predicted structure (blue). Structures were aligned using Chimera,[10] with an RMSD (Cα) of 0.525 Å between 97 pruned atom pairs (residues 1-5 of the N-term and residues 69-74 of the β-wing were not considered for RMSD calculation).

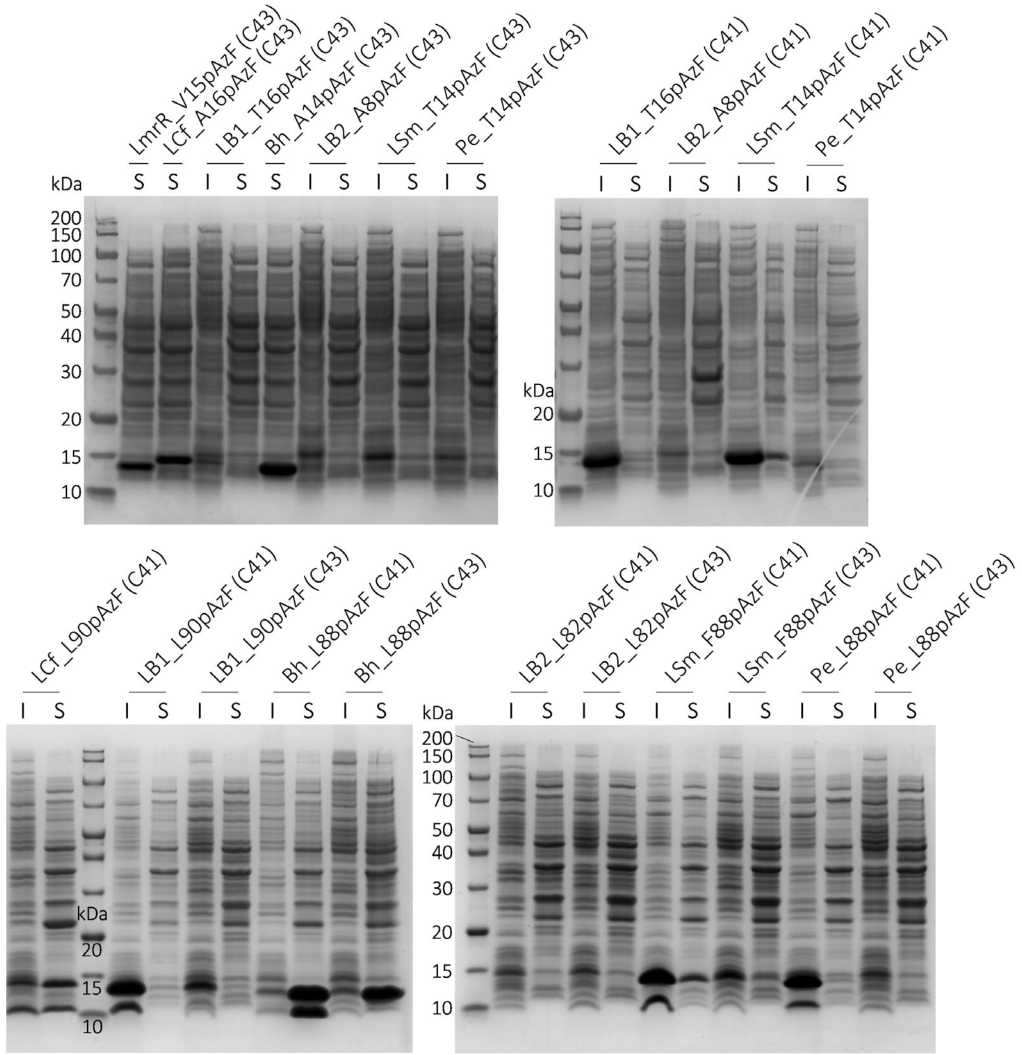

Figure S8. SDS-PAGE (12% polyacrylamide, Bis-Tris) of small-scale (4 mL) expressions of PadR proteins incorporating pAzF at the "V15X" or "M89X" positions using BL21(DE3) C43 (C43) or BL21(DE3) C41 (C41). Insoluble (I) and soluble (S) fractions after cell lysis were analyzed separately, loading ≈ 0.1 OD_{600} units per well. Ladder = Thermo Scientific PageRuler unstained broad-range protein ladder. Expected masses of target PadR proteins range from 13.0-15.6 kDa (monomer). For various "M89X" variants, a band can be observed below the target proteins that corresponds to truncated proteins as a results of translation termination instead of ncAA incorporation. For ncAA incorporation into LB1, LB2, LSm or Pe, either the protein did not express well or the protein was mainly present in the insoluble fraction, and cultivation at lower temperature did not improve this.

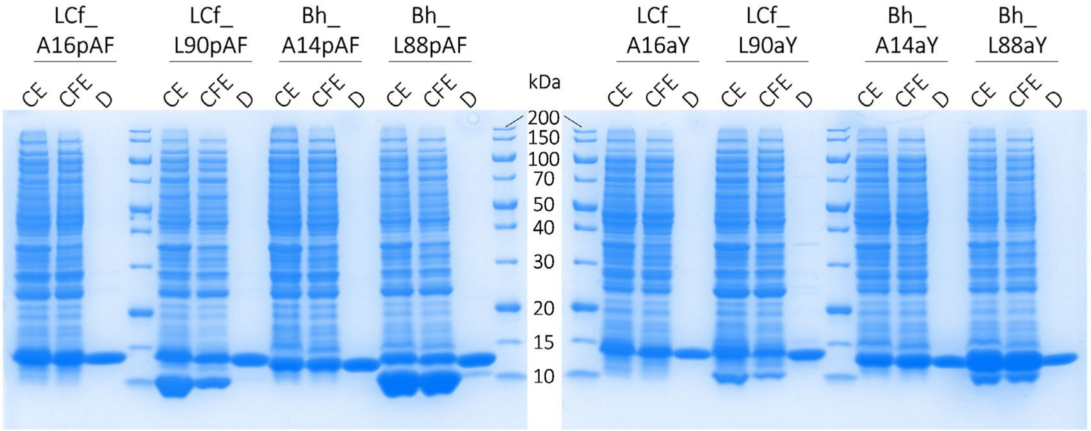

Figure S9. SDS-PAGE (12% polyacrylamide, Bis-Tris) of StrepTag purification of LCf/Bh incorporating pA(z)F or aY at the "V15X" or "M89X" positions. CE = cell extract, CFE = cell-free extract, D = desalted (and reduced in the case of pAF) purified protein. Samples of ≈ 0.1 OD_{600} units were loaded per well. For desalted pure protein, 7 µL of a 15 µM sample (dimer concentration) was loaded. Ladder = Thermo Scientific PageRuler unstained broad-range protein ladder. Expected masses of target PadR proteins range from 14.4-15.6 kDa (monomer). For "M89X" variants, a band is observed below the target proteins that corresponds to truncated proteins as a results of translation termination instead of ncAA incorporation.

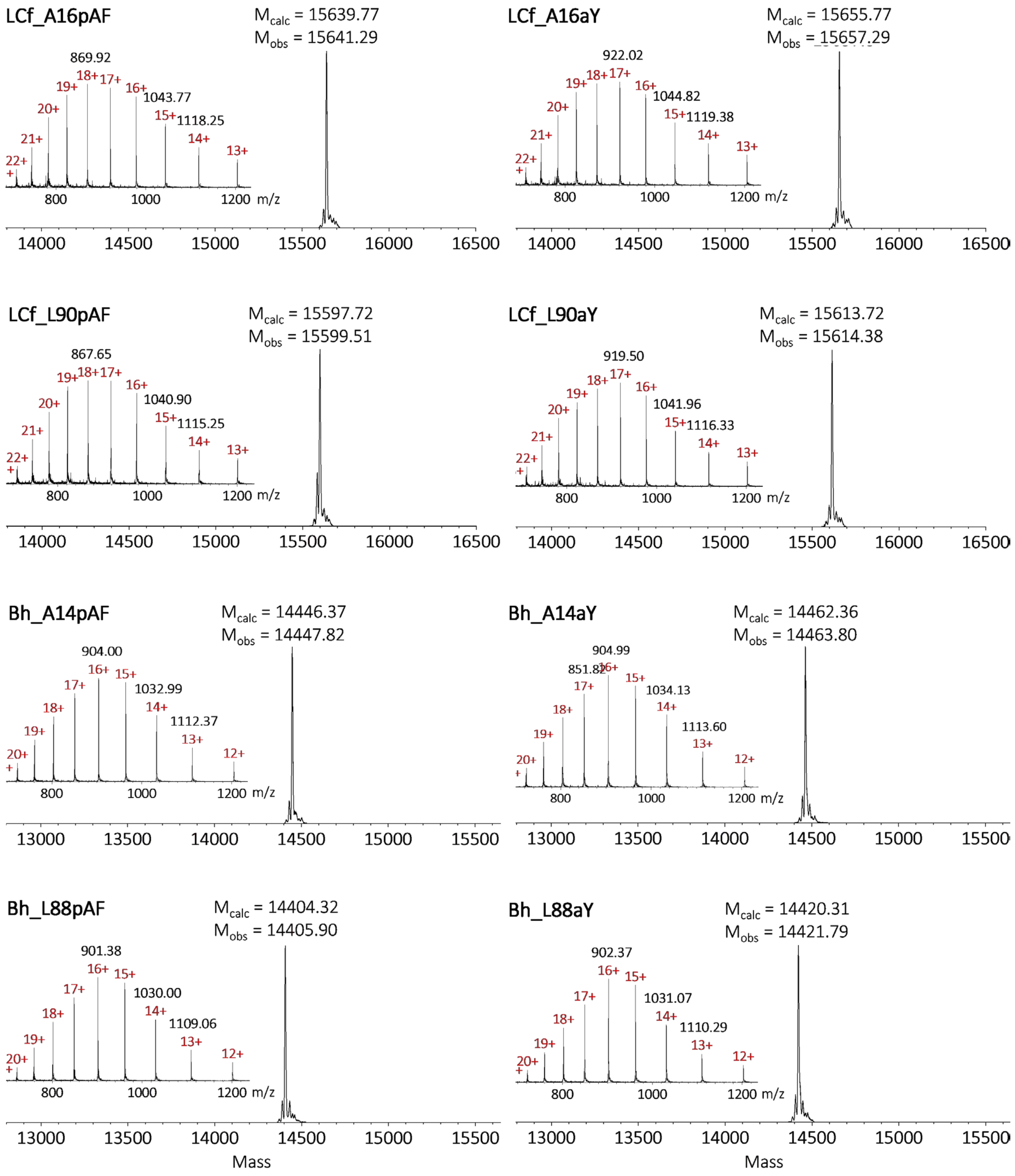

Figure S10. MS (ESI-QTOF) of purified PadR proteins with genetically incorporated pAF or aY, showing the obtained mass spectra and their deconvolution. For experimental details, see SI section 11.

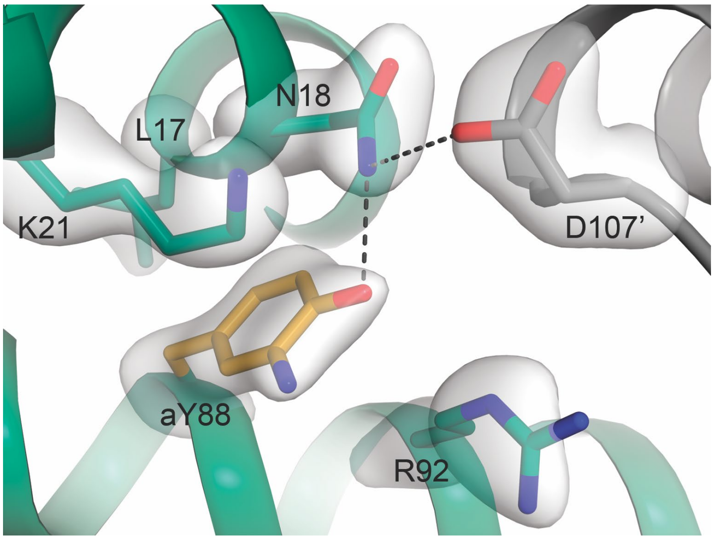

Figure S11. Zoomed-in view of one of the aY88 residues (orange) and surrounding amino acids in the Bh_L88aY crystal structure (PDB 9QBD). Shown in grey are the *Fo*-*Fc* omit densities of the depicted side chains (2.10 Å resolution, contoured at 3σ). Dashed lines depict hydrogen bonds.

4. Supporting Tables

Table S1. Apparent melting temperatures determined by thermofluor assay.

Protein	Buffer	$T_{m\text{-}app}$ (°C)
LmrR[a]	MOPS pH 7.0	-
LCf	MOPS pH 7.0	72
LB1	MOPS pH 7.0	54
Bh[b]	MOPS pH 7.0	75
LB2	MOPS pH 7.0	45
LSm	MOPS pH 7.0	49
Pe	MOPS pH 7.0	45
LmrR[a]	PBS pH 6.5	-
LmrR_V15pAF	PBS pH 6.5	66
LCf	PBS pH 6.5	75
LCf_A16pAF	PBS pH 6.5	74
LCf_L90pAF	PBS pH 6.5	59
Bh	PBS pH 6.5	73
Bh_A14pAF[c]	PBS pH 6.5	-
Bh_L88pAF	PBS pH 6.5	69
LmrR[a]	MES pH 5.5	-
LmrR_V15aY	MES pH 5.5	56
LCf	MES pH 5.5	58
LCf_A16aY	MES pH 5.5	55
LCf_L90aY	MES pH 5.5	43
Bh	MES pH 5.5	70
Bh_A14aY	MES pH 5.5	62
Bh_L88aY	MES pH 5.5	61

Results are the average of technical duplicates. [a]$T_{m\text{-}app}$ could not be determined due to a high starting fluorescence signal, which may be due to binding of the SYPRO orange dye in the pocket of LmrR before denaturation.[9] Interestingly, in contrast to LmrR, reliable $T_{m\text{-}app}$ values could be obtained for LmrR_V15pAF / V15aY, indicating introduction of ncAAs may lower the affinity for the SYPRO orange dye, leading to a more reliable melting curve. [b]Thermofluor experiments performed using 50 or 100 µM protein and 60X SYPRO orange. [c]$T_{m\text{-}app}$ could not be determined due to absence of a notable change in fluorescence intensity. See **Figure S5** for RFU and dRFU/dT curves, and SI section 9 for more details. NcAA incorporation is found to lower the $T_{m\text{-}app}$ of the proteins to various degrees, depending on the ncAA and the position of incorporation. Compared to LCf in PBS pH 6.5, incorporation of pAF into LCf at position A16 only results in a decrease in $T_{m\text{-}app}$ of 1 °C, whereas incorporation at position L90 leads to a decrease in $T_{m\text{-}app}$ of 16 °C. A similar trend can be observed for the incorporation of aY into LCf at these positions, where incorporation at position A16 results in a decrease in $T_{m\text{-}app}$ of 3 °C, and incorporation at position L90 leads to a decrease in $T_{m\text{-}app}$ of 15 °C compared to LCf in MES pH 5.5. Overall, this shows that incorporation of an ncAA at position L90 of LCf has a much larger effect on the $T_{m\text{-}app}$ than incorporation at position A16. This could indicate that the incorporation of the bulky aromatic ncAAs at position 90 has a larger effect on local packing and global conformation than incorporation at position A16 of the LCf protein, leading to a larger decrease in $T_{m\text{-}app}$. Although the $T_{m\text{-}app}$ of Bh_A14pAF could not be determined, a similar trend as seen for LCf is not observed for the incorporation of ncAAs into Bh. Incorporation of pAF into Bh at position L88 results in a decrease in $T_{m\text{-}app}$ of 4 °C compared to Bh in PBS pH 6.5, and incorporation of aY at positions A14 and L88 leads to a decrease in $T_{m\text{-}app}$ of 8 °C and 9 °C, compared to Bh in MES pH 5.5, respectively. Incorporation at position A14 and L88 thus seem to have a similar effect in Bh, both decreasing the $T_{m\text{-}app}$, but by not as much as observed for LCf at position L90.

Table S2. Summary of the crystallographic statistics.

	**BhPadR**	**BhPadR_L88aY**
**Data collection**		
Wavelength (Å)	1.5418	0.9655
Resolution range (Å)	44 – 2.05 (2.11 – 2.05)	111 – 2.10 (2.16 – 2.10)
Space group	$P4_12_12$	$P4_32_12$
Unit cell, a, b, c (Å)	48.3, 48.3, 107.7	51.6, 51.6, 222.8
R_{merge}	0.052 (1.072)	0.118 (1.576)
R_{meas}	0.056 (1.152)	0.122 (1.662)
R_{pim}	0.021 (0.416)	0.028 (0.508)
Number of observations	64366 (4861)	333803 (13149)
Number unique	8597 (646)	18462 (1318)
Mean(I/σI)	21.4 (2.0)	13.2 (1.2)
$CC_{1/2}$	1.000 (0.592)	0.999 (0.620)
Completeness (%)	100.0 (100.0)	99.0 (89.8)
Multiplicity	7.5 (7.5)	18.1 (10.0)
**Refinement**		
Resolution range (Å)	27 – 2.05	56 – 2.10
R_{work} / R_{free}	0.225 / 0.277	0.212 / 0.245
Content AU,	1 protein chain (residues 4-108)	2 protein chains (A, residues 6-108; B, residues 1-108)
nr of non-H atoms, protein, waters	873, 9, 0	1767, 48
Average B-factors (Å^2) protein, water	52.2, 52.3	48.4, 47.8
RMSD bond lengths (Å), angles (°)	0.008, 0.97	0.007, 0.89
Ramachandran favored, outliers (%)	99.0, 0.0	99.0, 0.0
Rotamers, outliers (%)	1.1	0.5
Molprobity-Clashscore	1.72	0.28
PDB entry	9QBC	9QBD

Values in parentheses refer to the highest resolution shell. AU, asymmetric unit.

Table S3. Control reactions without Cu^{II}Phen for the FC-alkylation of **2** with **1**.

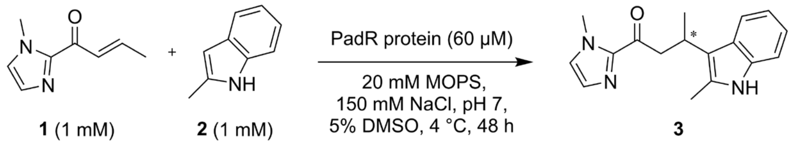

Entry	Catalyst	Yield (%)	ee (%)[a]
1	-	2 ± 1	< 1
2	LmrR	2 ± 0	2 ± 5
3	LCf	2 ± 0	< 1
4	LB1	2 ± 0	-1 ± 1
5	Bh	3 ± 2	< 1
6	LB2	2 ± 0	< 1
7	LSm	3 ± 1	< 1
8	Pe	2 ± 0	< 1

[a]ee is assigned relative to the enantiomer obtained with LmrR, with negative values representing the opposite enantiomer. Entries are based on at least three experiments, using two independently produced batches of protein. Errors are the standard deviation of the results.

Table S4. Results for the PadR_pAF / aY promoted FC-alkylation of **2** with **4**, and controls without ncAA.

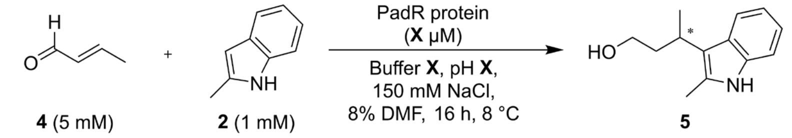

Entry	Catalyst	Catalyst conc. (µM)	pH[a]	Yield (%)	ee (%)[b]
1	-	-	6.5	< 1	n.d.
2	LmrR	20	6.5	2 ± 0	-1 ± 2
3[c]	LmrR_V15pAF*	20	6.5	53 ± 2	39 ± 2
4[d]	LCf	60	6.5	2 ± 0	5 ± 2
5	LCf_A16pAF	60	6.5	8 ± 0	11 ± 7
6	LCf_L90pAF	60	6.5	5 ± 0	5 ± 5
7[d]	Bh	60	6.5	7 ± 0	3 ± 1
8	Bh_A14pAF	60	6.5	12 ± 0	-12 ± 1
9	Bh_L88pAF	60	6.5	6 ± 1	1 ± 1
10	-	-	5.5	< 1	n.d.
11[c]	LmrR	60	5.5	5 ± 0	-6 ± 1
12[c]	LmrR_V15aY*	60	5.5	10 ± 1	-67 ± 1
13[d]	LCf	60	5.5	2 ± 0	4 ± 0
14	LCf_A16aY	60	5.5	15 ± 1	19 ± 3
15	LCf_L90aY	60	5.5	19 ± 1	< 1
16[d]	Bh	60	5.5	8 ± 0	-1 ± 1
17	Bh_A14aY	60	5.5	15 ± 1	-6 ± 1
18	Bh_L88aY	60	5.5	34 ± 3	-52 ± 3

Unless otherwise specified, results are based on at least three experiments, using two or more independently produced batches of protein. n.d. = not determined. [a]pH 6.5 = phosphate buffer (50 mM); pH 5.5 =MES buffer (20 mM). [b]ee is assigned relative to the enantiomer obtained with LmrR_V15pAF, with negative values representing the opposite enantiomer. [c]Data from Brouwer *et al.*[11] [d]Results are the average of technical triplicates. Errors are the standard deviation of the results.

5. General Information

All commercial reagents were used as received from vendors without further purification. aY was purchased from Merck (3-amino-*L*-tyrosine dihydrochloride monohydrate, 98%) and pAzF was purchased from Iris-Biotech (4-Azido-*L*-phenylalanine hydrochloride, 99%). 2-methylindole (> 99%) was purchased from TCI and crotonaldehyde was purchased from Merck (crotonaldehyde, mixture of *cis* and *trans* ~1:20, > 99.5%). Plasmid pEvol_pAzFRS.2.t1 was obtained from Addgene (plasmid #73546), and was a gift from Farren Isaacs.[12] Plasmid pEvol_*Mj*aYRS was obtained from Addgene (plasmid #153557), and was a gift from Huiwang Ai.[13] Primers were synthesized by Eurofins Genomics GmbH. Plasmid purification (QIAprep Spin Miniprep) and PCR purification (QIAquick) kits were purchased from QIAGEN. DNA sequencing was carried out by Eurofins Genomics GmbH. Standard recipes for Luria-Bertani (LB) medium (10 g/L bacto tryptone, 5 g/L yeast extract, 10 g/L NaCl and 15 g/L agar for LB-Agar plates) and 2xYT medium (16 g/L bacto tryptone, 10 g/L yeast extract, 5 g/L NaCl) were used. 1000X Antibiotic and inducer stock solutions were used and prepared as follows: ampicillin (100 mg/mL in MilliQ); chloramphenicol (34 mg/mL in 96% EtOH); *L*-arabinose (0.2 g/mL in MilliQ); isopropyl β-D-1-thiogalactopyranoside (IPTG) (1 M in MilliQ), and were sterilized through filtration using a 0.2 µm syringe filter (Whatman). Cultures were cultivated in a New Brunswick Innova 42 orbital shaker at a speed of 180 rpm (1 inch stroke). Sonication was performed with a Qsonica Q125 sonicator equipped with a 1/4" (6.4mm) probe. SDS-PAGE gels were stained using InstantBlue (abcam). Strep-Tactin resin (Strep-Tactin Superflow high capacity) and Desthiobiotin were purchased from IBA-Lifesciences. Phosphate-buffered saline (PBS) was prepared with 50 mM Na_2HPO_4 and 150 mM NaCl. DNA and protein concentrations were determined based on A_{260} and A_{280} values measured using a Thermo Scientific Nanodrop 2000 UV-Vis spectrophotometer. Concentrations of PadR proteins throughout the manuscript are reported as concentrations of dimeric protein. Vivaspin 20 10 kDa MWCO (Sartorius) and Amicon Ultra 0.5 mL 10 kDa MWCO (Millipore) centrifugal units were used for concentrating proteins. Analytical thin-layer chromatography (TLC) was carried out using Supelco silica gel 60 F_{254} plates. Visualization was accomplished with UV light. Purification of reactions was carried out by flash chromatography under positive pressure using Supelco silica gel (60 Å, 230-400 mesh, 40-63 µm). Nuclear magnetic resonance spectra (^{1}H and ^{13}C NMR experiments) were recorded on a Varian 400 MHz. Chemical shifts (δ) for proton and carbon are reported in parts per million (ppm) downfield from tetramethylsilane and are referenced to the proton or carbon resonance of residual $CHCl_3$ (δ_H = 7.26 ppm, δ_C = 77.0 ppm). NMR data are represented as follows: chemical shift (ppm), multiplicity (s = singlet, d = doublet, t = triplet, q = quartet, hept = heptet, m = multiplet), coupling constant in Hertz (Hz), and integration. HPLC analysis was performed using a Shimadzu Prominence LC-20AD equipped with a Shimadzu SPD-M20A diode array detector. SFC analysis was performed using a Waters Acquity UPC2 system. Mass spectrometry measurements were performed using a Waters Acquity H-class UPLC coupled to a Waters Xevo G2 QTOF. FPLC was performed using an Äkta Purifier system equipped with a UV-900 Detector. Chimera was used for visualization of protein structures.[10]

6. Cloning of PadR constructs

Codon optimized gene sequences for the six PadR candidates, including a C-terminal StrepTag and the homologous "K55D" and "K59Q" mutations known to decrease DNA-binding in LmrR, were ordered in pET17b vectors (Genscript), cloned between the NdeI and HindIII restriction sites. For Bh, only the mutation at homologous position "55" was introduced as the residue at homologous position "59" did not have a positively charged side chain. pET17b_LmrR_WT and pET17b_LmrR_V15X ("X" represents the amber stop codon, TAG), including mutations for decreased DNA-binding (K55D_K59Q), were available from previous work.[14] Plasmids were transformed into chemically competent *E. coli* NEB10β for storage or BL21(DE3) C41 or C43 for protein production. Competent cells were prepared according to the Inoue method.[15] 1-5 µL of isolated plasmid was added to 50 µL competent *E. coli*, followed by 30 min incubation on ice, a 45 s heat shock at 42 °C, and another 2 min incubation on ice directly after. Cells were then recovered in 0.7 mL SOC medium at 37 °C for ≈ 1 h in a thermomixer (750 rpm). The transformation mixture was subsequently spun down at 3000 xg and cells resuspended in 100-200 µL of residual volume. 20-100% of the cells was then plated on LB-agar plates containing ampicillin (100 µg/mL) and incubated at 37 °C overnight. Single colonies were inoculated into 5 mL LB medium containing ampicillin (100 µg/mL) and incubated at 37 °C in an orbital shaker overnight. Glycerol stocks were prepared by mixing 650 µL of cell culture with 350 µL of sterile 50% (v/v) glycerol and stored at -70 °C. PCRs to introduce mutations into the target genes were performed with *PfuTurbo* (Hotstart) DNA polymerase (2.5 U) (Agilent) using *Pfu* reaction buffer (1X), dNTPs (200 µM), forward primer (0.3 µM), reverse primer (0.3 µM), template plasmid DNA (5-25 ng), DMSO (3% v/v), and sterilized MilliQ water in a total reaction volume of 50 µL. The following PCR-protocol was used: (1) initial denaturation at 95 °C for 2 min, (2) 18 cycles of denaturation at 95 °C for 30 s, annealing at T_m - 5 °C (63-64 °C) for 30 s, and extension at 68 °C for 4 min, (3) final extension at 68 °C for 10 min. PCR products were then treated with restriction enzyme *DpnI* (20 U) (New England Biolabs) for 1-2 h at 37 °C, followed by purification using a QIAquick PCR purification kit and transformation of 1-5 µL of purified PCR product into NEB10β as described above. Plasmid DNA was isolated using a QIAprep Spin Miniprep kit and sent for sanger sequencing using T7 or T7-term primer to confirm correct mutation of the target gene. Isolated plasmid was then co-transformed together with pEVOL_pAZFRS2.t1 into competent BL21(DE3) C41 or C43, or transformed into competent BL21(DE3) C41 already harboring pEVOL_*Mj*aYRS. 50-100% of the transformation mixtures were plated on LB-agar plates containing ampicillin (100 µg/mL) and chloramphenicol (34 µg/mL), and incubated at 37 °C overnight. Single colonies were inoculated into 5 mL LB medium containing ampicillin (100 µg/mL) and chloramphenicol (34 µg/mL) and incubated at 37 °C in an orbital shaker overnight. Glycerol stocks were prepared by mixing 650 µL of cell culture with 350 µL of sterile 50% (v/v) glycerol and stored at -70 °C.

Gene sequences (DNA / protein)

Residues that were mutated in the PadR proteins to mimic the mutations introduced in LmrR that decrease DNA-binding ("K55D" and "K59Q" in LmrR) are depicted in blue. The C-terminal StrepTag for affinity purification is depicted in pink. The positions for ncAA incorporation, "V15X" and "M89X" relative to LmrR, and the corresponding codon that was changed to the amber codon (TAG) in the DNA sequence are depicted in green ("V15X") and orange ("M89X"). The N-terminal methionine of LmrR and LCf is generally cleaved off during protein production in *E. coli*. Note that, compared to the sequence of native LmrR (Uniprot A2RI36), the LmrR gene used in this work contains an additional glycine directly after methionine 1. To keep amino acid numbering consistent with native LmrR, numbering is started from the additional glycine.

LmrR

ATGGGTGCCGAAATCCCGAAAGAAATGCTGCGTGCTCAAACCAATGTCATCCTGCTGAATGTCCTGAAACAAGGCGATAACTATGTGTATGGCATTATC
AAACAGGTGAAAGAAGCGAGCAACGGTGAAATGGAACTGAATGAAGCCACCCTGTATACGATTTTTGATCGTCTGGAACAGGACGGCATTATCAGCTCT
TACTGGGGTGATGAAAGTCAAGGCGGTCGTCGCAAATATTACCGTCTGACCGAAATCGGCCATGAAAACATGCGCCTGGCGTTCGAATCCTGGAGTCG
TGTGGACAAAATCATTGAAAATCTGGAAGCAAACAAAAAATCTGAAGCGATCAAATCTAGAGGTGGCAGCGGTGGCTGGAGCCACCCGCAGTTCGAAA
AATAA

~~M~~GAEIPKEMLRAQTNVILLNVLKQGDNYVYGIIKQVKEASNGEMELNEATLYTIFDRLEQDGIISSYWGDESQGGRRKYYRLTEIGHENMRLAFESWSRVDKIIE
NLEANKKSEAIKSRGGSGGWSHPQFEK*

LCf

ATGGCGGAGGAAATCAGCAAGGACATGATTCGTGGTCACATGGATGCGGTGATCCTGAACATTCTGAGCCAGGCGGACAGCTACGGCTATGAAGTGA
GCAAAACCGTTAAGAAACTGAGCGAGAACCAATACGAGATCAACGAAGCGACCCTGTATACCGTTTTCGACCGTCTGGAGCAGAGCGGTCACATTGAA
AGCTACTGGGGCGATGAGAGCCAAGGTGGCCGTCGTAAGTACTATAAAATCACCGCGACCGGTCTGGAAAAGCTGGCGAACGCGCGTGATAGCTGGC
AGTTCGCGCAAAAGATCATTACCAAACTGATCATGGGTACCATTGAAAACAAGGGCGACGATAGCTGGAAAATTTATGGTGGCAGCGGTGGCAGCGCG
TGGAGCCACCCGCAGTTTGAGAAATAA

~~M~~AEEISKDMIRGHMDAVILNILSQADSYGYEVSKTVKKLSENQYEINEATLYTVFDRLEQSGHIESYWGDESQGGRRKYYKITATGLEKLANARDSWQFAQKIIT
KLIMGTIENKGDDSWKIYGGSGGSAWSHPQFEK*

LB1

ATGAAGCCGGAGATTAGCAAAGAAACCATCCGTGGTCACACCACCACCATCGTGCTGAACATTCTGAACCAGGGTGACAGCTACGGCTATGCGATCGC
GAAGACCATTAAAACCCTGAGCCACGCGGCGTACGATATTAACGAGGCGACCCTGTATACCGTTTTCGACCGTCTGGAACAGAACGGTGATATCACCA
GCTATTGGGGTAACGAGACCCAGGGTGGCCGTCGTAAGTACTATCAAATTAGCGCGCAGGGTCAAGCGACCCTGCAACACAACATCGACGAATGGCA
GTTCGCGAAACAAGTGATCGACGATCTGATTCTGGGCCGTATCGATGAACATGAAGGTGGCAGCGGTGGCAGCGCGTGGAGCCACCCGCAATTTGAG
AAGTAA

MKPEISKETIRGHTTTIVLNILNQGDSYGYAIAKTIKTLSHAAYDINEATLYTVFDRLEQNGDITSYWGNETQGGRRKYYQISAQGQATLQHNIDEWQFAKQVIDD
LILGRIDEHEGGSGGSAWSHPQFEK*

Bh
ATGAACATCGAAAGCGACATCATTCGTGGTCACATTGATGCGGTGGTTCTGAACTTCCTGAAGGACAACGATAGCTACGGCTATGAGCTGAGCAAGCT
GATCACCGACAAAACCAACGGCGAGTACGAAATCAACGGCCAGACCCTGTATAGCGCGATTGACCGTCTGGAGAGCAAGAAACTGATCGAAGGTTACT
GGGGCGATGAGAGCCAAGGTGGCCGTCGTAAGTACTATCGTATTACCGAGGAAGGTAAGAAATTCCTGAAAGAGGAACGTGATATCTGGCTGTTTACC
AAGAAAATCATTGACAAGCTGCTGGATATTGAAGGTGGCAGCGGTGGCAGCGCGTGGAGCCACCCGCAGTTTGAGAAATAA

MNIESDIIRGHIDAVVLNFLKDNDSYGYELSKLITDKTNGEYEINGQTLYSAIDRLESKKLIEGYWGDESQGGRRKYYRITEEGKKFLKEERDIWLFTKKIIDKLLDI
EGGSGGSAWSHPQFEK*

LB2
ATGGTGCGTGGCTACCTGAGCGCGATCGTGCTGAACGTTCTGAGCCAAGGTGCGAGCTACGGCTATCAGGTTACCAAGGATATCAACGCGCTGAGCG
GTGGCGCGTACGTGATTAACGAGGCGACCCTGTATACCGTTTTCGACCGTCTGGAACAAGCGGGTAGCATCACCGGTTTTTGGGGCGATGAGAGCCA
AGGTGGCCGTCGTAAATACTATCAGATCACCGACGCGGGCCAGCAACAGCTGATTCAGGAGCGTGCGACCTGGAAGCTGGCGAAAACCACCCTGGAA
GCGCTGATTAACGGTAACAACGGTGGCAGCGGTGGCAGCGCGTGGAGCCACCCGCAATTCGAAAAGTAA

MVRGYLSAIVLNVLSQGASYGYQVTKDINALSGGAYVINEATLYTVFDRLEQAGSITGFWGDESQGGRRKYYQITDAGQQQLIQERATWKLAKTTLEALINGN
NGGSGGSAWSHPQFEK*

LSm
ATGGACATCAGCAAGGATCTGATTCGTGGCCACACCGATACCATCATTCTGAACATCCTGAGCCAGGGTGACAGCTACGGCTATCAAGTGAGCAAGAG
CATTCGTCAGCTGAGCAGCAAACAATACGAGCTGAACGAAGCGACCCTGTATACCGCGTTCGACCGTCTGGAGCAGGTTGGTGATATCCGTAGCTATT
GGGGTGACCAGACCCAAGGTGCGCGTCGTAAATACTATACCCTGACCGATCAGGGCCAAGAGCACTTCAAGAGCGCGCAGCAAGAATGGGACTTTGC
GAAAGATATCATTAGCCACCTGATCAGCGGTCGTATTAAGGACTATGATGAACCGGGTGGCAGCGGTGGCAGCGCGTGGAGCCACCCGCAATTTGAAA
AATAA

MDISKDLIRGHTDTIILNILSQGDSYGYQVSKSIRQLSSKQYELNEATLYTAFDRLEQVGDIRSYWGDQTQGARRKYYTLTDQGQEHFKSAQQEWDFAKDIISH
LISGRIKDYDEPGGSGGSAWSHPQFEK*

Pe
ATGCAGATCAGCAAGGACCTGATTCGTGGTAACACCGATACCATCATTCTGAACCTGCTGAACCAGCAAGATAGCTACGGCTATCAGATCGCGAAGCAA
GTGAAATACCTGAGCAACGACGCGTATGAGATTAACGAAGCGACCCTGTACACCGTTTTCGACCGTCTGGAGCAGCACCAAGATATCGAAAGCTATTG
GGGTGACGAGACCCAGGGTGCGCGTCGTAAATACTATCGTATTACCGAAACCGGTCAGCAACTGCTGGCGAGCAACGTGCAGCAATGGGATTTCAGC
AAGAAAATCATTGAGAAGCTGATCAAAGGCAGCATTGCGTACGACAAGCATGGTGGCAGCGGTGGCAGCGCGTGGAGCCACCCGCAATTTGAAAAATA
A

MQISKDLIRGNTDTIILNLLNQQDSYGYQIAKQVKYLSNDAYEINEATLYTVFDRLEQHQDIESYWGDETQGARRKYYRITETGQQLLASNVQQWDFSKKIIEKLI
KGSIAYDKHGGSGGSAWSHPQFEK*

Primer list (5' to 3')

Primer name	Primer sequence
LCf_A16X_fw	CGTGGTCACATGGATTAGGTGATCCTGAACATTC
LCf_A16X_rev	GAATGTTCAGGATCACCTAATCCATGTGACCACG
LB1_T16X_fw	CGTGGTCACACCACCTAGATCGTGCTGAACATTC
LB1_T16X_rev	GAATGTTCAGCACGATCTAGGTGGTGTGACCACG
Bh_A14X_fw	CGTGGTCACATTGATTAGGTGGTTCTGAACTTCC
Bh_A14X_rev	GGAAGTTCAGAACCACCTAATCAATGTGACCACG
LB2_A8X_fw	CGTGGCTACCTGAGCTAGATCGTGCTGAACG
LB2_A8X_rev	CGTTCAGCACGATCTAGCTCAGGTAGCCACG
LSm_T14X_fw	CGTGGCCACACCGATTAGATCATTCTGAACATCC
LSm_T14X_rev	GGATGTTCAGAATGATCTAATCGGTGTGGCCACG
Pe_T14X_fw	CGTGGTAACACCGATTAGATCATTCTGAACCTGC
Pe_T14X_rev	GCAGGTTCAGAATGATCTAATCGGTGTTACCACG
LCf_L90X_fw	CCGGTCTGGAAAAGTAGGCGAACGCG
LCf_ L90X _rev	CGCGTTCGCCTACTTTTCCAGACCGG
LB1_ L90X _fw	CAGGGTCAAGCGACCTAGCAACACAACATCGACG
LB1_ L90X _rev	CGTCGATGTTGTGTTGCTAGGTCGCTTGACCCTG
Bh_ L88X _fw	CCGAGGAAGGTAAGAAATTCTAGAAAGAGGAACG
Bh_ L88X _rev	CGTTCCTCTTTCTAGAATTTCTTACCTTCCTCGG
LB2_ L82X _fw	GGGCCAGCAACAGTAGATTCAGGAGCGTGCG
LB2_ L82X _rev	CGCACGCTCCTGAATCTACTGTTGCTGGCCC
LSm_ F88X _fw	GGCCAAGAGCACTAGAAGAGCGCGCAGCAAG
LSm_ F88X _rev	CTTGCTGCGCGCTCTTCTAGTGCTCTTGGCC
Pe_ L88X _fw	GGTCAGCAACTGTAGGCGAGCAACGTGCAGC
Pe_ L88X _rev	GCTGCACGTTGCTCGCCTACAGTTGCTGACC

Mutated nucleotides are underlined

7. Protein Production and Purification

Protein expression

A tube with 5 mL LB containing appropriate antibiotics (100 µg/mL ampicillin for pET17b and 34 µg/mL chloramphenicol for pEvol) was inoculated from a glycerol stock of *E. coli* BL21(DE3) C41 or C43 cells harboring a pET17b_PadR variant, and in the case of ncAA incorporation also pEvol_pAzFRS2.t1 or pEvol_*Mj*aYRs, and incubated overnight at 37 °C in an orbital shaker. The dense pre-culture was 100-fold diluted in fresh culture media (LB or 2xYT for proteins without ncAA; LB for proteins with ncAA) containing the same antibiotics in a non-baffled Erlenmeyer flask five times the size in volume compared to the used media. The culture was incubated at 37 °C in an orbital shaker with moderate shaking (180 rpm) until an OD_{600} of ≈ 0.8-1.0, at which point expression was induced by addition of IPTG (1 mM), and in the case of ncAA incorporation also *L*-arabinose (0.02%) and pAzF (1 mM, as a solid) or aY (2 mM, as a solid). The culture was further incubated overnight (18-24 h) at 30 °C in an orbital shaker with moderate shaking (180 rpm). Cells were collected by centrifugation in 50 mL centrifuge tubes (8000 xg, 10 min, 4 °C) or swing buckets (3428 xg, 45 min, 4 °C) and cell pellets frozen and stored at -21 °C. LmrR, LCf, LB1, Bh, LSm, LCf_A16pAzF and Bh_A14pAzF were produced in BL21(DE3) C43. LB2, Pe, LmrR_V15pAzF, LCf_L90pAzF, Bh_L88pAzF, LmrR_V15aY, LCf_A16aY, LCf_L90aY, Bh_A14aY and Bh_L88aY were produced in BL21(DE3) C41. Small-scale protein expressions were conducted similar as described above, but with main cultures of 4 mL in tubes, induced with a master mix containing inducers and ncAA in LB with appropriate antibiotics. Samples of these small-scale cultures were spun down and redissolved in Bugbuster primary amine-free (Millipore) for cell lysis. Lysed cells were spun down again and the supernatant (soluble fraction) and pellet (insoluble fraction) analyzed by SDS-PAGE (**Figure S8**).

Protein purification

Cell pellets of 100-200 mL culture media were thawed and resuspended in 10-15 mL cold PBS (pH 8.0, sterilized), containing protease inhibitor (c0mplete EDTA-free, 1 pill/50 mL (Roche) + 2 mM $MgCl_2$) or EDTA (1 mM). Resuspended cells were placed in an ice-bath and lysed by sonication (5-10 min, 5 sec on, 7 sec off, amplitude 70%). Cell extracts (CE) were centrifuged (18514 xg, 30-40 min, 4 °C) to obtain the cell-free extracts (CFE). CFE was passed through a 0.45 µm syringe filter and loaded onto a gravity column containing ≈ 3-4 mL Strep-Tactin Superflow high-capacity resin equilibrated in PBS pH 8.0. The resin was then washed with sterilized PBS pH 8.0 (5 column volumes (CV) total) and the target protein eluted with the same buffer containing 5 mM desthiobiotin (3 CV total). Fractions containing protein were pooled and concentrated using a 10 kDa MWCO centrifugal filter. Purified protein was exchanged into catalysis buffer by dialysis (6-8 MWCO (Spectra/Por), ≈2 mL protein / 1L buffer, 2x >7 h at 8 °C) or by using PD-10 Sephadex G-25 M desalting columns (Cytiva), and subsequently frozen in liquid nitrogen and stored at -21 °C. *Observation*: it was found that using 500 mM instead of 150 mM NaCl for storing PePadR improved its stability. For PadR_pAF variants, prior to desalting, pAzF was reduced to pAF by adding TCEP (90 mM in PBS pH 8) to a final concentration of 10 mM, and subsequently incubated overnight at 8 °C on a turning platform. Protein concentrations were determined based on A_{280} values measured using a Nanodrop and molar extinction coefficients (ε_{280}) calculated for each PadR variant. Extinction coefficients were approximated using the ProtParam Expasy web server https://web.expasy.org/protparam/, and corrected for the absorbance of noncanonical amino acids (ε_{280} aY = 2342 M^{-1} cm^{-1}, ε_{280} pAF = 1333 M^{-1} cm^{-1}).[11,14] The identity and purity of purified proteins were confirmed by MS (ESI-QTOF, **Figures S3** and **S10**). For several variants, the purification process was followed by SDS-PAGE (**Figures S2** and **S9**). Protein yields ranged from 20-150 mg/L culture.

8. Size Exclusion Chromatography

Size exclusion chromatography was performed using a Superdex 75 increase 10/300 GL column (Cytiva) mounted on an Äkta Purifier (**Figure S3**). Samples containing purified PadR protein (1.7-2.0 mg/mL, in 20 mM MOPS, 150 mM NaCl, pH 7.0) were loaded using a 100 µL loop and subsequently eluted using 20 mM MOPS, 150 mM NaCl, pH 7 buffer as eluent (0.8 mL/min). A calibration curve was made by running proteins from the gel filtration LMW calibration kit (GE Healthcare). Expected elution volumes (Ve_{calc}) for homodimeric proteins were calculated using the calibration curve and compared to observed elution volumes (Ve_{obs}).

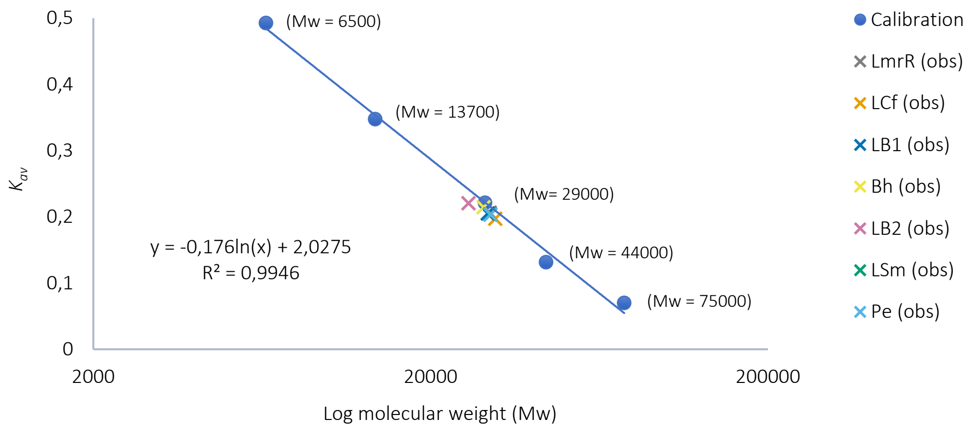

$$K_{av} = \frac{Ve - V0}{Vc - V0} \quad \rightarrow \quad Ve = K_{av}(Vc - V0) + V0$$

$K_{av} = partition\ coefficient$

$Ve = elution\ volume$

$V0 = void\ volume\ (7.86\ mL)$

$Vc = geometric\ column\ volume\ (24\ mL)$

Protein	Mw (dimer)	Ve_{calc} (mL)	Ve_{obs} (mL)
LmrR	29942	11.3	11.2
LCf	31099	11.2	11.0
LB1	29525	11.3	11.2
Bh	28712	11.4	11.3
LB2	25909	11.7	11.4
LSm	30027	11.3	11.2
Pe	30083	11.3	11.1

9. Thermofluor Assay

The fluorescence-based thermofluor assay was used to determine $T_{m\text{-app}}$ values.[16] Unless otherwise specified, samples were prepared by mixing diluted commercial SYPRO orange solution (Invitrogen 5000X, final concentration of 15X), purified protein (final concentration of 50 µM) and buffer up to a total volume of 20 µL in a clear 96-well PCR plate. The plates were sealed, and heated from 20 to 95 °C in a CFX96 real-time PCR machine (Bio-Rad) with a linear gradient of increasing temperature (1 °C/30 sec). The temperature at the maximum rate of fluorescence change (dRFU/dT) was taken as $T_{m\text{-app}}$, see **Figure S5** and **Table S1** for results. Fluorescence intensities for Bh were low, but could be increased by using higher protein and SYPRO orange final concentrations (100 µM and/or 60X, respectively). For Bh_A14pAF, $T_{m\text{-app}}$ could not be determined by thermofluor assay due the absence of a notable change in fluorescence intensity. Measurements of LmrR (without ncAA incorporated) were found to exhibit high starting fluorescence intensities, which may be due to binding of the SYPRO orange dye in the pocket of LmrR before denaturation.[9] This resulted in abnormal melting peaks in the dRFU/dT plots, and therefore, the $T_{m\text{-app}}$ of LmrR samples were not determined. A $T_{m\text{-app}}$ of 65 °C for LmrR is reported in the literature.[17] Interestingly, in contrast to LmrR, we were able to obtain reliable $T_{m\text{-app}}$ values for LmrR_V15pAF / V15aY. It could be that introduction of these ncAAs lower the affinity for the SYPRO orange dye, leading to a more reliable melting curve.

10. Protein Crystallography

Prior to crystallization, BhPadR and BhPadR_L88aY were further purified by size exclusion chromatography on a Superdex 75 increase 10/300 GL (Cytiva) using 20 mM HEPES, 150 mM NaCl, pH 7.0 as eluent. Crystallization conditions were identified with the sitting-drop vapor diffusion method, using the PACT premier, JCGS Plus, and Morpheus HT screens (Molecular Dimensions), and the PEG/Ion and Index HT screens (Hampton Research). Drops of 200 nL were dispensed using a Mosquito robot (SPTLabTech), mixing protein solution (~11 mg/mL in 20 mM HEPES, 150 mM NaCl, pH 7.0) with reservoir solutions at two volume ratios (75:125 and 125:75) in MRC 96-well 2-drop crystallization plates. Crystals grew at various conditions within a few days at 21 °C. For BhPadR, sitting drop crystallizations were subsequently set up using a 1:1 mixture of protein (1 µL, 11.3 mg/mL) and crystallization buffer (1 µL), based on JCSG Plus condition E4 (0.2 M lithium sulfate, 0.1 M Tris pH 8.5, and 1.26 M ammonium sulfate), varying the concentration of ammonium sulfate between 0.8-1.75 M. Crystals producing the best quality X-ray diffraction data were obtained with 1.4 M ammonium sulfate. Before flash-cooling, crystals were cryo-protected using a solution containing 0.2 M lithium sulfate, 0.1 M Tris pH 8.5 and 1.5 M ammonium sulfate, supplemented with 25% (v/v) PEG400. For BhPadR_L88aY, crystals were picked directly from the sitting-drop crystallizations in the MRC2 plates. Crystals were cryo-protected by brief transfer to a solution similar to the respective crystallization condition, supplemented with 25% (v/v) PEG 400 or glycerol, and subsequently flash cooled in liquid nitrogen. The crystal that gave the best quality X-ray diffraction data for BhPadR_L88aY was obtained with a crystal grown at crystallization condition H7 from the Index screen (0.15 M DL-malic acid pH 7.0, 20% (w/v) PEG 3350), using 11.4 mg/mL protein, and was cryo-protected using a solution containing 0.15 M malonate pH 7, 150 mM NaCl, 22% (w/v) PEG 3350 and 25% (v/v) glycerol.

X-ray diffraction data for BhPadR were collected in-house using Cu Kα radiation from a Bruker Microstar rotating-anode generator equipped with Helios mirrors. For BhPadR_L88aY, X-ray diffraction data were collected at the MASSIF-1 beamline of the ESRF synchrotron, Grenoble. Data indexing and integration were performed using XDS[18] or Xia2/DIALS[19], with final scaling and merging completed via Aimless[20] from the CCP4 software suite[21]. Initial phases and structural models were determined by molecular replacement with PHASER,[22] using a monomeric AlphaFold structure as a search model. Structures were subsequently refined through iterative rounds of manual model building in Coot,[23] interspersed with restrained refinement in REFMAC5.[24] Geometry restraints for 3-aminotyrosine (residue code TY2) were obtained from the CCP4 monomer library. The final rounds of refinement were performed with Phenix.refine.[25] Both structures were refined with isotropic B-factors including TLS parameterization. Validation of the final structures was conducted with MolProbity[26] and the wwPDB Validation Server (https://validate.wwpdb.org). Data collection and refinement statistics are summarized in **Table S2**. Coordinates and structure factors were deposited at the PDB with entry codes 9QBC and 9QBD for BhPadR and BhPadR_L88aY, respectively.

11. Protein Mass Spectrometry

UPLC-MS (ESI-QTOF) measurements were performed for purified proteins to verify identity and purity (**see Figures S3** and **S10**). Protein samples (0,05-0,15 mg/mL in 50:50 PBS pH 8.0 or catalysis buffer:MilliQ) were injected (5 µL) on an Acquity UPLC Protein BEH C4, 300 Å, 1.7 µm, 2.1 mm x 150 mm column using water (A) and acetonitrile (B) with 0.1% (v/v) formic acid as mobile phase at a flow rate of 0.3 mL/min using the following method: 90% A for 2 min, linear gradient to 50% A in 8 min, linear gradient to 5% A in 1 min, 5% A for 2 min, linear gradient back to 90% A in 0.1 min followed by 3.9 min of re-equilibration at 90% A. The total ion chromatograms obtained from mass spectrometry measurements were extracted to get the m/z spectra, which were subsequently deconvoluted using MagTran 1.0.[27] For calculation of expected protein masses, the protein molecular formula, obtained from the ProtParam Expasy web server, was corrected for the presence of a ncAA and used as input for an isotope simulation performed with Xcalibur Freestyle 1.8 using a profile resolution of 100000. The highest intensity isotope from this simulation was used as calculated mass (M_{calc}) for purified proteins. Accuracy of the ESI-QTOF instrument varied, at times leading to higher differences between the M_{calc} and observed mass (M_{obs}). Generally, ΔM_{calc}-M_{obs} varied between 0-5 Da, which was accurate enough to confirm the identity of the different protein variants. For LmrR_V15pAF and LmrR_V15aY see Brouwer *et al.*[11]

12. Catalysis and Workup Procedures

Cu^{II}-promoted FC-alkylation

Reactions were performed in a total volume of 300 μL in a 2 mL microcentrifuge tube. Catalysis buffer (20 mM MOPS, 150 mM NaCl, pH 7.0) and purified protein (desalted into catalysis buffer, 60 μM final concentration) were added to a total volume of 280 μL. Tubes were stored on ice and 10 μL of a freshly prepared Cu^{II}(1,10-phenanthroline)$(NO_3)_2$ stock solution (1.35 mM in DMSO:catalysis buffer 50:50, final concentration 45 μM) was added and the mixture incubated on ice for 30 min. Reactions were started by addition of 5 μL of a freshly prepared stock solution of substrate **1** (60 mM in DMSO, final concentration 1 mM) and 5 μL of a freshly prepared stock solution of substrate **2** (60 mM in DMSO, final concentration 1 mM), and the reaction tubes subsequently continuously inverted for 48 hours at 4 °C in a cold room. Afterwards, 20 μL of internal standard stock solution (2-phenylquinoline, 5 mM in acetonitrile) was added and the reaction mixtures extracted with ethyl acetate. For extraction, 0.6 mL of ethyl acetate was added and the tubes were subsequently heavily mixed (> 1 min) using a vortex equipped with an adapter for multiple microcentrifuge tubes. Mixtures were then spun down (1 min, 17000 xg) and the organic layer (500 μL) pipetted into a new microcentrifuge tube. This process was repeated with another 0.6 mL of ethyl acetate and the pooled extracts were dried by addition of a small spatula tip of Na_2SO_4. After moderate mixing on a vortex (> 1 min), insoluble material was spun down (5 min, 17000 xg) and the supernatant (800 μL) pipetted into a new microcentrifuge tube. Solvent was then removed *in vacuo* using an Eppendorf concentrator plus (45-60 min, 45 °C, V-HV), and the obtained residue redissolved in 150 μL of an *n*-heptane:isopropanol mixture (4:1, HPLC grade) by vortex mixing. Samples were then analyzed by normal-phase HPLC to determine yield and ee (Chiralpak AD-H, 88:12 *n*-heptane:iPrOH, 1 mL/min, 30 min).

Iminium-promoted FC-alkylation

Reactions were done the same as described in previous work.[11] Reactions were performed in a total volume of 300 μL in a 2 mL microcentrifuge tube. Catalysis buffer and purified protein (desalted into catalysis buffer, 60 μM final concentration unless otherwise specified) were added to a total volume of 276 μL, and mixtures were stored on ice. PBS (50 mM Na_2HPO_4, 150 mM NaCl, pH 6.5) was used as catalysis buffer for variants harboring pAF, and MES buffer (20 mM MES, 150 mM NaCl, pH 5.5) was used as catalysis buffer for variants harboring aY. Reactions were started by addition of 12 μL of a freshly prepared stock solution of substrate **4** (125 mM in DMF, final concentration 5 mM) and 12 μL of a freshly prepared stock solution of substrate **2** (25 mM in DMF, final concentration 1 mM), and the reaction tubes subsequently incubated in a thermomixer (750 rpm) for 16 hours at 8 °C in a cold room. Afterwards, 12 μL of internal standard stock solution (3-(3-hydroxypropyl)indole, 5 mM in DMF) and 60 μL of a freshly prepared reduction solution ($NaBH_4$, 20 mg/mL in 0.5% w/v NaOH) were added and the tubes mixed for another 30 min at 8 °C. Reaction mixtures were extracted by addition of *n*-butanol (400 μL), followed by heavy mixing (> 1 min) using a vortex equipped with an adapter for multiple microcentrifuge tubes. Mixtures were subsequently spun down (5 min, 17000 xg) and the organic layer (300 μL) pipetted into a new microcentrifuge tube containing a small spoon tip of Na_2SO_4. Tubes were then moderately mixed on a vortex (> 1 min) and insoluble material spun down (5 min, 17000 xg). The supernatant (150 μL) was analyzed by SFC to determine yield and ee (Chiracel OJ-3, scCO2 (A) and MeOH (B) as mobile phase, linear gradient from 3% B to 50% B in 4.5 min, followed by 50% B for 2 min, 1.8 mL/min).

13. Synthetic Procedures and Characterization

Substrate 1 ((E)-1-(1-methyl-1H-imidazol-2-yl)but-2-en-1-one)

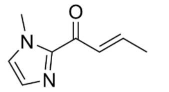

Prepared following reported literature procedure.[28,29] Compound **1** (0.57 g, 3.8 mmol; 25% yield) was obtained as a yellow gum from N-methylimidazole (2.5 mL, 31.4 mmol, 2.1 equiv) and crotonic acid (1.3 g, 15.1 mmol, 1.0 equiv). The spectroscopic data were consistent with those reported in the literature.[28,29] **^{1}H NMR** (400 MHz, $CDCl_3$) δ 7.42 (dq, *J* = 15.5, 1.7 Hz, 1H), 7.17 (s, 1H), 7.18 – 7.07 (m, 1H), 7.04 (s, 1H), 4.04 (s, 3H), 1.98 (dd, *J* = 7.0, 1.5 Hz, 3H). **^{13}C NMR** (101 MHz, $CDCl_3$) δ 180.7, 144.2, 143.7, 129.1, 128.0, 127.1, 36.4, 18.6. **HRMS** (ESI-MS) m/z: $[M\text{-}H]^+$ calcd for $C_8H_{10}N_2O$ 151.0864, found 151.0866.

Reference product 3 (1-(1-methyl-1H-imidazol-2-yl)-3-(2-methyl-1H-indol-3-yl)butan-1-one)

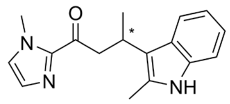

Prepared following reported literature procedure.[30] Compound **3** (49 mg, 0.17 mmol; 55% yield) was obtained as a dark red solid from **1** (48 mg, 0.32 mmol, 1.0 equiv) and 2-methylindole (**2**, 92 mg, 0.70 mmol, 2.2 equiv). The spectroscopic data were consistent with those reported in the literature.[30–32] **^{1}H NMR** (400 MHz, $CDCl_3$) δ 7.72 – 7.61 (m, 2H), 7.25 – 7.17 (m, 1H), 7.11 (s, 1H), 7.09 – 7.00 (m, 2H), 6.94 (s, 1H), 3.85 (s, 3H), 3.83 – 3.72 (m, 1H), 3.70 – 3.54 (m, 2H), 2.43 (s, 3H), 1.47 (d, *J* = 7.1 Hz, 3H). **^{13}C NMR** (101 MHz, $CDCl_3$) δ 192.4, 143.4, 135.4, 130.5, 128.9, 127.5, 126.7, 120.7, 119.4, 119.0, 115.6, 110.3, 46.4, 36.1, 27.2, 21.2, 12.4. **HRMS** (ESI-MS) m/z: $[M\text{-}H]^-$ calcd for $C_{17}H_{18}N_3O$ 280.1456, found 280.1455. **HPLC** Chiralpak AD-H (*n*-heptane:iPrOH 88:12, 1 mL/min), t_R = 13.3-13.6 min; 19.4-19.7 min.

Cu^{II}(1,10-phenanthroline)(NO_3)$_2$ (Cu^{II}Phen) was prepared following the reported literature procedure.[33,34]

14. Calibration Curves and HPLC Chromatograms

A calibration curve for product **3** (Cu^{II}-promoted FC-alkylation) was made using synthesized reference compound (see SI section 13). For product **5** (iminium-promoted FC-alkylation), calibration data from Brouwer *et al* was used.[11] Samples were prepared similar as when setting up the catalysis reactions, but without catalyst and with 0.1-1.0 mM reference product **3**, or 0.1-1.1 mM reference product **5** instead of substrates. Workup and analysis were then done the same as would be done for catalysis reactions. Retention times sometimes slightly shifted over time, but authenticity of the products, substrates and internal standard was confirmed by their UV-vis absorbance. For each of these, their general retention time and characteristic maximum absorbance (λ_{max}, not taking into account absorbance < 225 nm) are stated. For product **3**, ee is assigned relative to the enantiomer obtained with LmrR, with negative values representing the opposite enantiomer. For product **5**, ee is assigned relative to the enantiomer obtained with LmrR_V15pAF, with negative values representing the opposite enantiomer. Examples of HPLC and SFC traces are shown below.

<u>**HPLC – Product 3** (1-(1-methyl-1H-imidazol-2-yl)-3-(2-methyl-1H-indol-3-yl)butan-1-one)</u>

HPLC method: Chiralpak AD-H, 88:12 *n*-heptane:iPrOH, 1 mL/min, 30 min, 20 µL injection, integrated at 274 nm.

IS: 4.4-4.5 min (λ_{max} 251 nm)
1+**2**: 5.2-5.3 min (λ_{max} (**1**) 271 nm; λ_{max} (**2**) 294 nm)
3 "+": 13.3-13.6 min (λ_{max} 275 nm)
3 "-": 19.4-19.7 min (λ_{max} 275 nm)

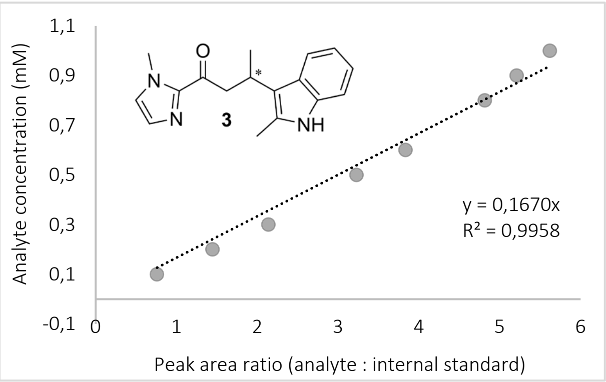

Reference product 3

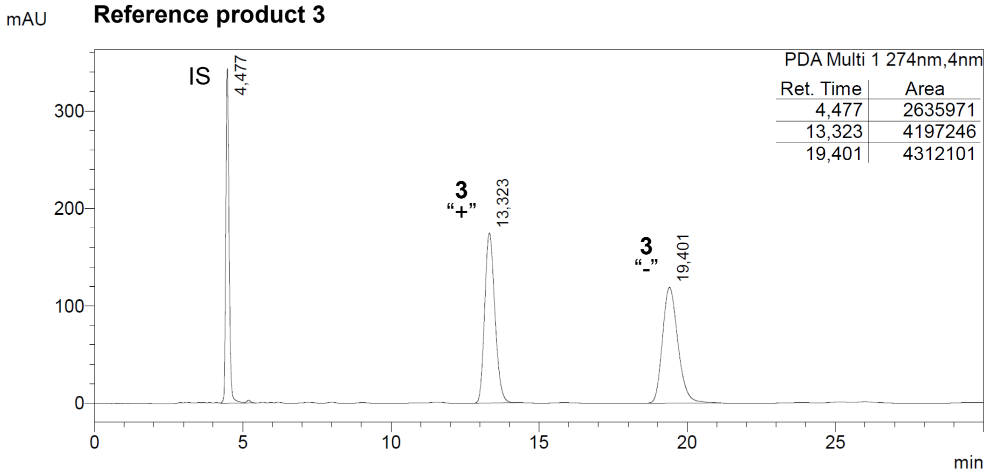

LmrR-Cu^{II}Phen

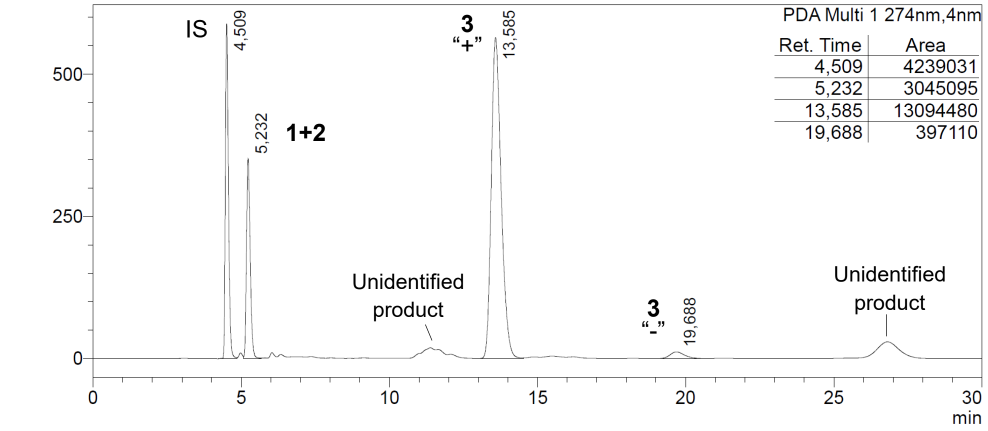

LB2-Cu^{II}Phen

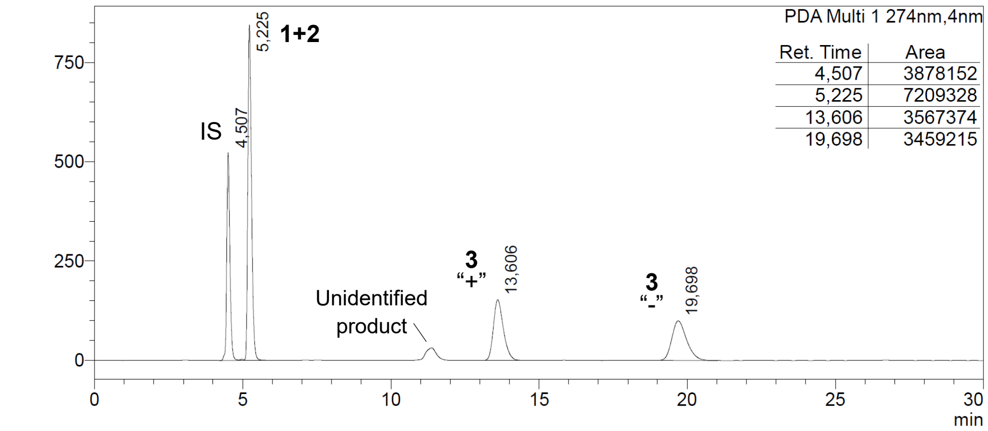

SFC – product 5 (3-(2-methyl-1H-indol-3-yl)butan-1-ol)

SFC Method: Chiralcel OJ-3, $scCO_2$ (A) and MeOH (B) as mobile phase, linear gradient from 3% B to 50% B in 4.5 min, followed by 50% B for 2 min (6.5 min total), column was re-equilibrated at 3% B for 1.5 min in between samples (not shown in SFC-traces), 1.8 mL/min, 10 µL injection, integrated at 282 nm.

5 "+": 2.7 min (λ_{max} 282 nm)
5 "-": 2.9 min (λ_{max} 282 nm)
IS: 3.5 min (λ_{max} 281 nm)
2: 3.6 min (λ_{max} 269 nm)

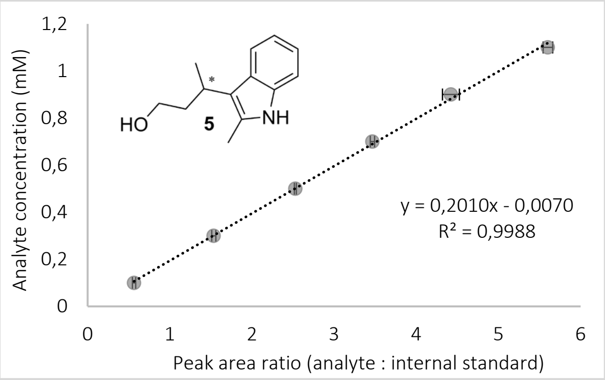

Reference product 6 [11]

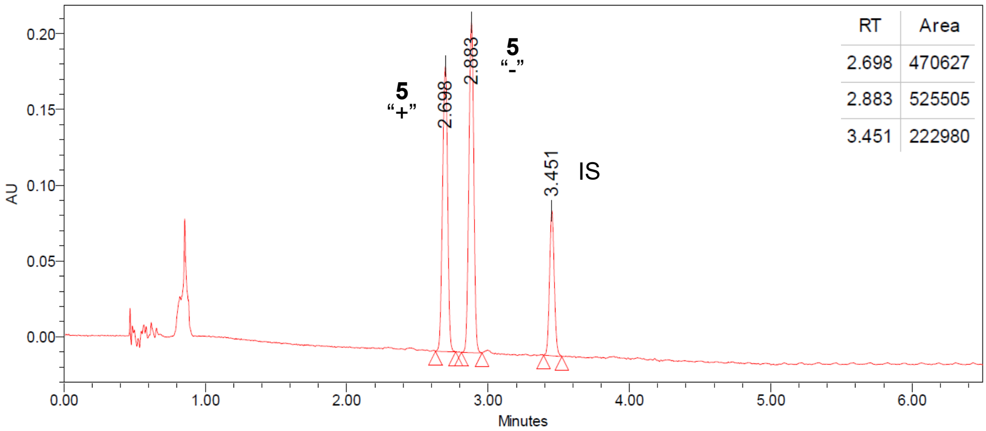

V15pAF (20 µM, pH 6.5) [11]

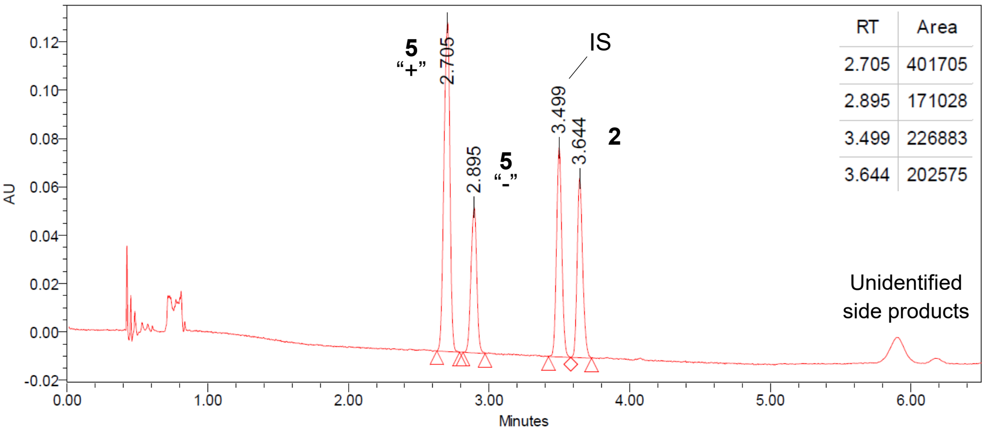

Bh_L88aY (60 µM, pH 5.5)

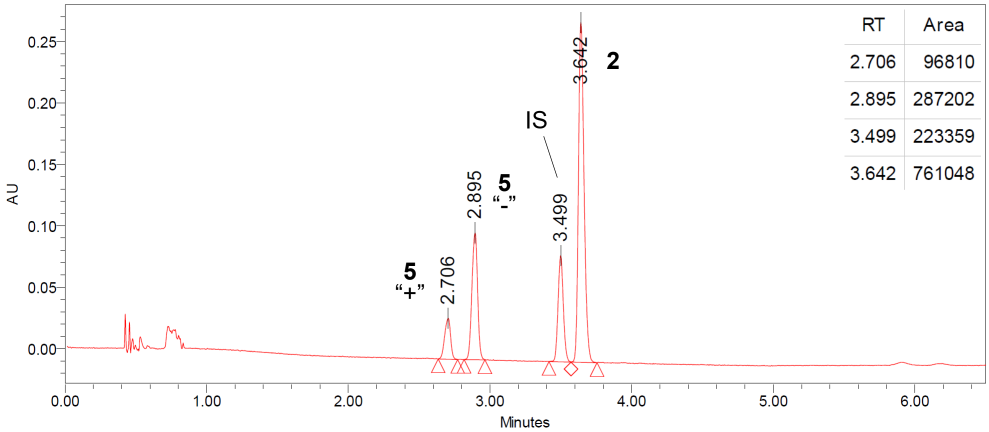

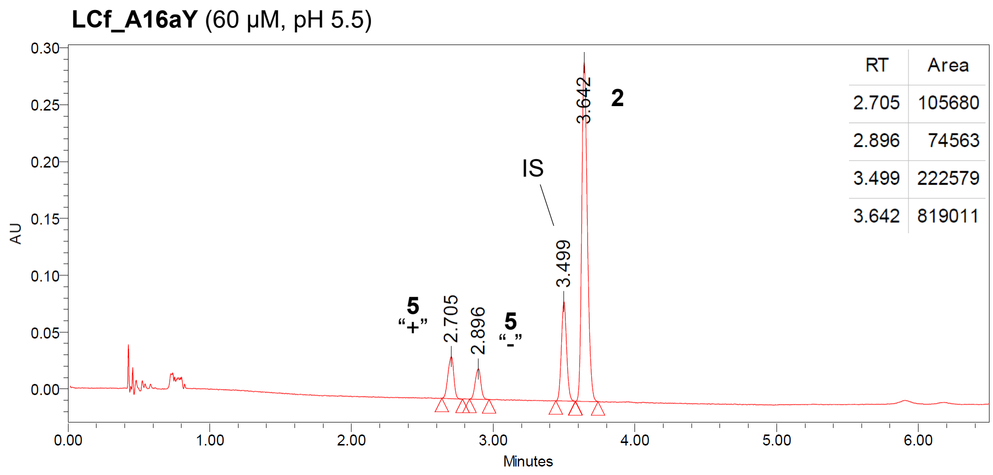

15. References

[1] S. F. Altschul, W. Gish, W. Miller, E. W. Myers, D. J. Lipman, *J. Mol. Biol.* **1990**, *215*, 403.

[2] K. Katoh, J. Rozewicki, K. D. Yamada, *Brief. Bioinform.* **2019**, *20*, 1160.

[3] F. Sievers, D. G. Higgins, *Protein Sci.* **2018**, *27*, 135.

[4] A. M. Waterhouse, J. B. Procter, D. M. A. Martin, M. Clamp, G. J. Barton, *Bioinformatics* **2009**, *25*, 1189.

[5] I. Letunic, P. Bork, *Nucleic Acids Res.* **2021**, *49*, W293.

[6] J. Jumper, R. Evans, A. Pritzel, T. Green, M. Figurnov, O. Ronneberger, K. Tunyasuvunakool, R. Bates, A. Žídek, A. Potapenko, A. Bridgland, C. Meyer, S. A. A. Kohl, A. J. Ballard, A. Cowie, B. Romera-Paredes, S. Nikolov, R. Jain, J. Adler, T. Back, S. Petersen, D. Reiman, E. Clancy, M. Zielinski, M. Steinegger, M. Pacholska, T. Berghammer, S. Bodenstein, D. Silver, O. Vinyals, A. W. Senior, K. Kavukcuoglu, P. Kohli, D. Hassabis, *Nature* **2021**, *596*, 583.

[7] R. Evans, M. O'Neill, A. Pritzel, N. Antropova, A. Senior, T. Green, A. Žídek, R. Bates, S. Blackwell, J. Yim, O. Ronneberger, S. Bodenstein, M. Zielinski, A. Bridgland, A. Potapenko, A. Cowie, K. Tunyasuvunakool, R. Jain, E. Clancy, P. Kohli, J. Jumper, D. Hassabis, *BioRxiv Prepr.* **2022**, 10.1101/2021.10.04.463034.

[8] E. Krieger, K. Joo, J. Lee, J. Lee, S. Raman, J. Thompson, M. Tyka, D. Baker, K. Karplus, *Proteins Struct. Funct. Bioinforma.* **2009**, *77*, 114.

[9] S. Boivin, S. Kozak, R. Meijers, *Protein Expr. Purif.* **2013**, *91*, 192.

[10] E. F. Pettersen, T. D. Goddard, C. C. Huang, G. S. Couch, D. M. Greenblatt, E. C. Meng, T. E. Ferrin, *J. Comput. Chem.* **2004**, *25*, 1605.

[11] B. Brouwer, F. Della-Felice, A.-M. W. H. Thunnissen, G. Roelfes, *Chem. Sci.* **2025**, *16*, 8721.

[12] M. Amiram, A. D. Haimovich, C. Fan, Y.-S. Wang, H.-R. Aerni, I. Ntai, D. W. Moonan, N. J. Ma, A. J. Rovner, S. H. Hong, N. L. Kelleher, A. L. Goodman, M. C. Jewett, D. Söll, J. Rinehart, F. J. Isaacs, *Nat. Biotechnol.* **2015**, *33*, 1272.

[13] S. Zhang, H. Ai, *Nat. Chem. Biol.* **2020**, *16*, 1434.

[14] I. Drienovská, C. Mayer, C. Dulson, G. Roelfes, *Nat. Chem.* **2018**, *10*, 946.

[15] H. Im, *Bio-101* **2011**, *1*, e143.

[16] U. B. Ericsson, B. M. Hallberg, G. T. DeTitta, N. Dekker, P. Nordlund, *Anal. Biochem.* **2006**, *357*, 289.

[17] A. Gran-Scheuch, S. Hanreich, I. Keizer, J. W. Harteveld, E. Ruijter, I. Drienovská, *Faraday Discuss.* **2024**, *252*, 279.

[18] W. Kabsch, *Acta Crystallogr. D Biol. Crystallogr.* **2010**, *66*, 125.

[19] J. Beilsten-Edmands, G. Winter, R. Gildea, J. Parkhurst, D. Waterman, G. Evans, *Acta Crystallogr. Sect. Struct. Biol.* **2020**, *76*, 385.

[20] P. R. Evans, G. N. Murshudov, *Acta Crystallogr. D Biol. Crystallogr.* **2013**, *69*, 1204.

[21] J. Agirre, M. Atanasova, H. Bagdonas, C. B. Ballard, A. Baslé, J. Beilsten-Edmands, R. J. Borges, D. G. Brown, J. J. Burgos-Mármol, J. M. Berrisford, P. S. Bond, I. Caballero, L. Catapano, G. Chojnowski, A. G. Cook, K. D. Cowtan, T. I. Croll, J. É. Debreczeni, N. E. Devenish, E. J. Dodson, T. R. Drevon, P. Emsley, G. Evans, P. R. Evans, M. Fando, J. Foadi, L. Fuentes-Montero, E. F. Garman, M. Gerstel, R. J. Gildea, K. Hatti, M. L. Hekkelman, P. Heuser, S. W. Hoh, M. A. Hough, H. T. Jenkins, E. Jiménez, R. P. Joosten, R. M. Keegan, N. Keep, E. B. Krissinel, P. Kolenko, O. Kovalevskiy, V. S. Lamzin, D. M. Lawson, A. A. Lebedev, A. G. W. Leslie, B. Lohkamp, F. Long, M. Malý, A. J. McCoy, S. J. McNicholas, A. Medina, C. Millán, J. W. Murray, G. N. Murshudov, R. A. Nicholls, M. E. M. Noble, R. Oeffner, N. S. Pannu, J. M. Parkhurst, N. Pearce, J. Pereira, A. Perrakis, H. R. Powell, R. J. Read, D. J. Rigden, W. Rochira, M. Sammito, F. Sánchez Rodríguez, G. M. Sheldrick, K. L. Shelley, F. Simkovic, A. J. Simpkin, P. Skubak, E. Sobolev, R. A. Steiner, K. Stevenson, I. Tews, J. M. H. Thomas, A. Thorn, J. T. Valls, V. Uski, I. Usón, A. Vagin, S. Velankar, M. Vollmar, H. Walden, D. Waterman, K. S. Wilson, M. D. Winn, G. Winter, M. Wojdyr, K. Yamashita, *Acta Crystallogr. Sect. Struct. Biol.* **2023**, *79*, 449.

[22] A. J. McCoy, R. W. Grosse-Kunstleve, P. D. Adams, M. D. Winn, L. C. Storoni, R. J. Read, *J. Appl. Crystallogr.* **2007**, *40*, 658.
[23] P. Emsley, B. Lohkamp, W. G. Scott, K. Cowtan, *Acta Crystallogr. D Biol. Crystallogr.* **2010**, *66*, 486.
[24] G. N. Murshudov, P. Skubák, A. A. Lebedev, N. S. Pannu, R. A. Steiner, R. A. Nicholls, M. D. Winn, F. Long, A. A. Vagin, *Acta Crystallogr. D Biol. Crystallogr.* **2011**, *67*, 355.
[25] P. V. Afonine, R. W. Grosse-Kunstleve, N. Echols, J. J. Headd, N. W. Moriarty, M. Mustyakimov, T. C. Terwilliger, A. Urzhumtsev, P. H. Zwart, P. D. Adams, *Acta Crystallogr. D Biol. Crystallogr.* **2012**, *68*, 352.
[26] C. J. Williams, J. J. Headd, N. W. Moriarty, M. G. Prisant, L. L. Videau, L. N. Deis, V. Verma, D. A. Keedy, B. J. Hintze, V. B. Chen, S. Jain, S. M. Lewis, W. B. Arendall III, J. Snoeyink, P. D. Adams, S. C. Lovell, J. S. Richardson, D. C. Richardson, *Protein Sci.* **2018**, *27*, 293.
[27] Z. Zhang, A. G. Marshall, *J. Am. Soc. Mass Spectrom.* **1998**, *9*, 225.
[28] D. A. Evans, K. R. Fandrick, H.-J. Song, *J. Am. Chem. Soc.* **2005**, *127*, 8942.
[29] E. Benedetti, N. Duchemin, L. Bethge, S. Vonhoff, S. Klussmann, J.-J. Vasseur, J. Cossy, M. Smietana, S. Arseniyadis, *Chem. Commun.* **2015**, *51*, 6076.
[30] M. Bersellini, G. Roelfes, *Org. Biomol. Chem.* **2017**, *15*, 3069.
[31] C. Gutiérrez de Souza, M. Bersellini, G. Roelfes, *ChemCatChem* **2020**, *12*, 3190.
[32] I. Drienovská, A. Rioz-Martínez, A. Draksharapu, G. Roelfes, *Chem. Sci.* **2015**, *6*, 770.
[33] G. Roelfes, A. J. Boersma, B. L. Feringa, *Chem. Commun.* **2006**, *6*, 635.
[34] M. Navarro, E. J. Cisneros-Fajardo, A. Sierralta, M. Fernández-Mestre, P. Silva, D. Arrieche, E. Marchán, *JBIC J. Biol. Inorg. Chem.* **2003**, *8*, 401.
